# A modular 3D printed microfluidic system: a potential solution for continuous cell harvesting in large-scale bioprocessing

**DOI:** 10.1186/s40643-022-00550-2

**Published:** 2022-06-06

**Authors:** Lin Ding, Sajad Razavi Bazaz, Mahsa Asadniaye Fardjahromi, Flyn McKinnirey, Brian Saputro, Balarka Banerjee, Graham Vesey, Majid Ebrahimi Warkiani

**Affiliations:** 1 School of Biomedical Engineering, University of Technology Sydney, Sydney, NSW 2007 Australia; 2 School of Engineering, Macquarie University, Sydney, NSW 2109 Australia; 3 Regeneus Ltd, Paddington, Sydney, NSW 2021 Australia; 4 Institute of Molecular Medicine, Sechenov University, Moscow, 119991 Russia

**Keywords:** Microfluidics, Modular microfluidic system, Mesenchymal stem cells, 3D printing, Bioprocessing

## Abstract

Microfluidic devices have shown promising applications in the bioprocessing industry. However, the lack of modularity and high cost of testing and error limit their implementation in the industry. Advances in 3D printing technologies have facilitated the conversion of microfluidic devices from research output to applicable industrial systems. Here, for the first time, we presented a 3D printed modular microfluidic system consisting of two micromixers, one spiral microfluidic separator, and one microfluidic concentrator. We showed that this system can detach and separate mesenchymal stem cells (MSCs) from microcarriers (MCs) in a short time while maintaining the cell’s viability and functionality. The system can be multiplexed and scaled up to process large volumes of the industry. Importantly, this system is a closed system with no human intervention and is promising for current good manufacturing practices.

**Graphical Abstract:**

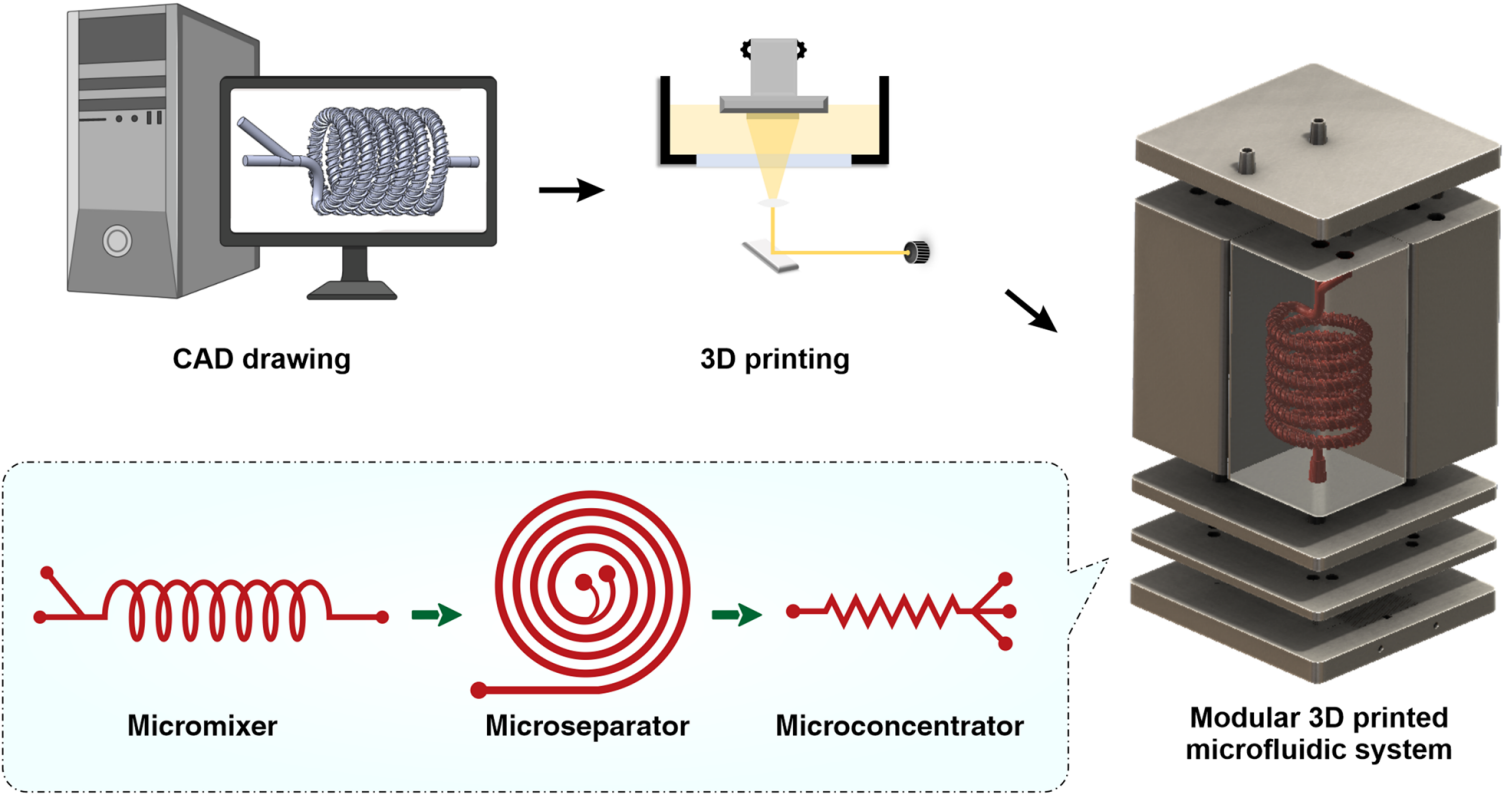

**Supplementary Information:**

The online version contains supplementary material available at 10.1186/s40643-022-00550-2.

## Introduction

Microfluidics, a science of precise fluid handling within the network of channels, has shown great promise in manipulating cells and particles. Microfluidics has attracted significant attention in biology and medical research due to their unique features including low price, high throughput, high customisability, and energy-efficiently compared to other technologies (Wang and Dandy 2017; Figeys and Pinto 2000). For example, micromixers have been used in chemicals synthesis and microparticle coating (Vasilescu et al. 2020). Multiple microfluidic devices, especially spiral microfluidic channels, have been demonstrated to separate or concentrate cells based on particle sizes (Xiang et al. 2019; Nivedita et al. 2017).

To date, microfluidic devices are widely used in laboratories but one of the major limitations for applying microfluidics in the industry is its customisability (Yi-Qiang et al. 2018). For instance, in the stem cell bioprocessing industry, each company has its own manufacturing protocol. The lack of standard procedure is one of the reasons for the low yield of cell products and the inconsistent clinical outcome of stem cell therapy (Jossen et al. 2018; Schnitzler et al. 2016). Although microfluidic devices have been applied in the stem cell bioprocessing industry as cell separator and concentrator in a labour-free, low-cost, and high-throughput manner (Moloudi et al. 2018, 2019), the lack of modularity and integrity makes them hard to be applied in the bioprocessing industry. Microfluidic devices are normally made from polydimethylsiloxane (PDMS) by soft lithography. Compiling these single microfluidic devices together to increase the throughput requires multiple external tubing and diverters to meet the industrial need, and testing and modifying them to meet the demand requires a huge amount of time and effort. 3D printing technology can be a good solution for this inadequacy. In recent years, the advances in 3D printing technologies have made it increasingly appealing for producing microfluidic devices (Bhattacharjee et al. 2016). The resolution of 3D printing allows direct construction of microfluidic channels with micrometre-level features, and the study and treatment of 3D printed resin enable the production of soft-lithography mould in a few hours (Vasilescu et al. 2020; Razavi Bazaz et al. 2019). Although 3D printing technologies are not the solution for large-scale manufacturing of microfluidic devices, their potential to modify changes and fabricate microfluidic devices in a few hours is unique and valuable for the industry. This feature hugely decreases the cost and time needed for rapid prototyping and building integrated microfluidic systems.

In the stem cell industry, microcarriers (MC)-based culture systems are a promising candidate for maximising cell manufacturing on a large scale. MCs facilitate massive cell expansion at a lower cost and allow control of cell culture parameters in a homogenous condition to produce consistent quality cell products at a large scale (Fardjahromi et al. 2020; Chen et al. 2020). Despite the enormous advantages of microcarrier-based technologies in maximising cell production, harvesting cells from MCs still faces challenges with high product quality and yield (Chen et al. 2013). The common method for harvesting is detaching cells with digestive enzymes and separating them from MCs using membrane-based filtration or centrifugation (Chilima et al. 2018; Tavassoli et al. 2018). Membrane-based filtration separates the cells with a physical porous filter. Clogging filters is the major limitation of this method (Schnitzler et al. 2016; Zydney 2016). In addition, membrane fouling has been shown to cause cell death, cell fate changes, and reduce the therapeutic potential of harvested cells (Chilima et al. 2018; Zydney 2016; Rodrigues et al. 2018). Centrifugation-based methods, particularly continuous flow centrifugation, are another alternative method for separating cells from MCs (Schnitzler et al. 2016). The advantage of this method is that it washes cells during separation, but the centrifugation process is time-consuming, potentially causing cell damage (Joseph et al. 2016). In addition, the continuous washing and centrifuging process cost more reagents and disposables (Serra et al. 2018). Hence, a continuous, clogging-free, highly efficient, and low-cost harvesting method is severely lacking in this area.

Herein, in this paper we report an integrated 3D printed modular microfluidic system containing two micromixers, one spiral separator, and one zig-zag concentrator. We used this system to detach and separate mesenchymal stem cells (MSCs) from MCs and eventually concentrate them in a smaller volume for downstream processing. At first, each module was characterised using cells and microbeads in different volume fractions and flow rates to obtain the optimum condition for the MSC harvesting. Then, the viability, proliferation, and therapeutic properties of MSCs harvested with our proposed integrated system were compared with the manual method, i.e., Millipore filtration. The results indicate that the developed microfluidic device is a promising candidate for automated MSCs harvesting and concentrating from MCs. In the end, we demonstrated that the system could be multiplexed to process samples with higher throughput.

## Materials and methods

### Device fabrications

For the fabrication of microfluidic devices using additive manufacturing, different techniques exist. Fused Deposition Modelling (FDM), Stereolithography (SLA), Digital Light Processing (DLP), two-photon polymerisation (2PP), Multijet, and wax printing are all capable of fabricating microfluidic devices. For the creation of complex microfluidic devices, however, DLP and wax printing methods show more promise in this regard. The fabrication process of these two methods is illustrated in Additional file 1: Fig. S1. The wax 3D printing method is a multi-step process, and the printed microfluidics are inherently fragile and prone to fault and error. As an alternative, DLP method has been selected for the current study because of its accuracy, precision, fast turn-around time, and the ability to fabricate robust complex microfluidic channels (Chai et al. 2021; Ding et al. 2022). Design selection consideration is introduced in detail in Additional file 1: Section S1.

The micromixers were designed in Solidworks 2018 × 64 edition (SolidWorks Corporation, USA) and fabricated with a high-resolution DLP resin printer (MiiCraft II, Hsinchu, Taiwan), with the layer thickness of 50 µm. BV-007 resin was used, which is an acrylate-based resin containing 80–95% acrylate components and 10–15% photoinitiator and additives (Razavi Bazaz et al. 2020a). After printing, the micromixers were carefully removed from the build plate, washed with isopropyl alcohol, and dried by air nozzle. This process was repeated three times to prevent uncured resin from clogging the channels. Then, the micromixers were cured by 450 nm UV light in a UV-curing chamber. The design and dimension of the micromixer are shown in Additional file 1: Fig. S2.

**Fig. 1:**
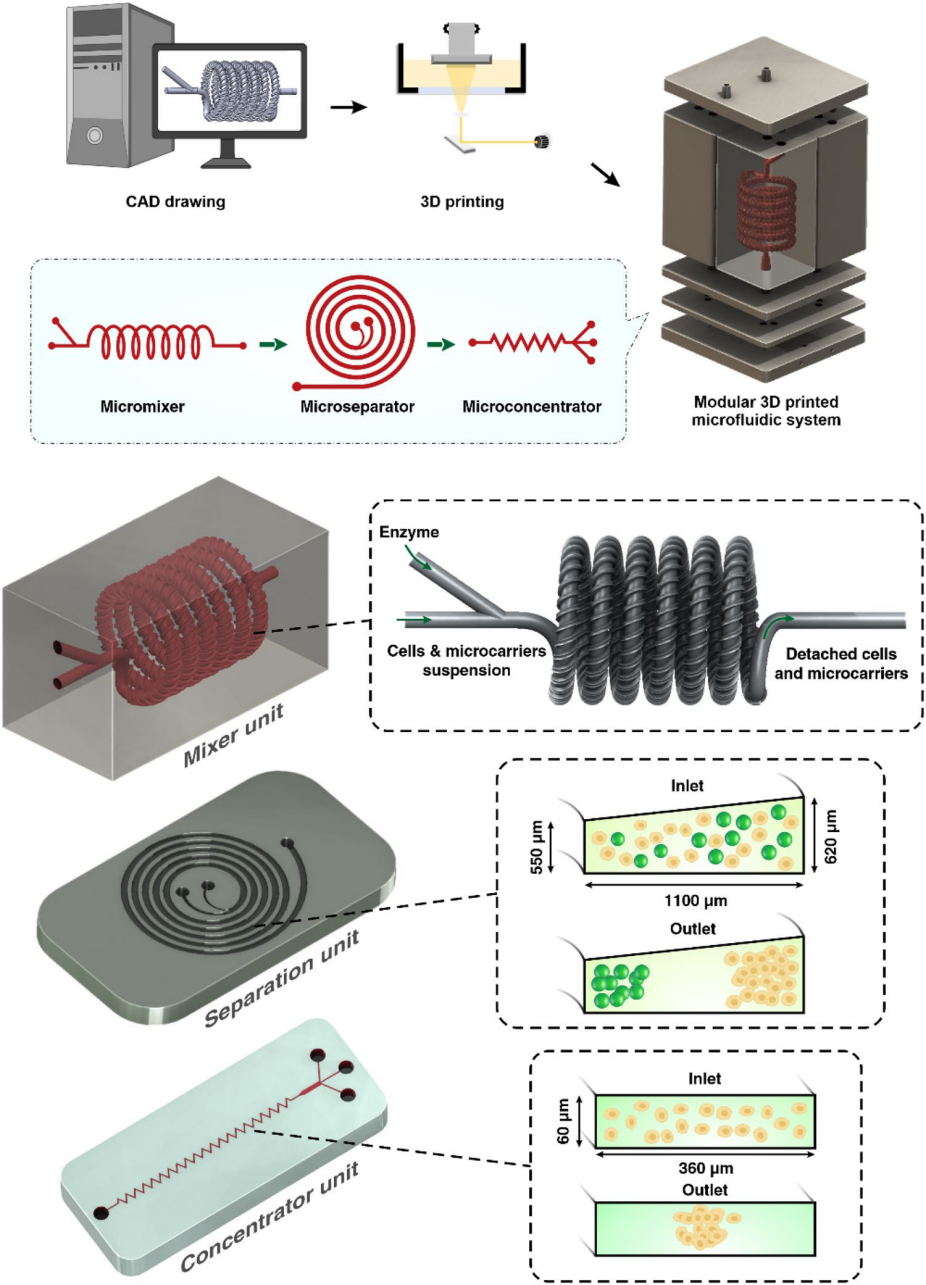
Schematic representation of the modular microfluidic system. The whole system was built by 3D printing technology. The system comprises two micromixers, a micro separator, and a zig-zag channel connected vertically to detach cells from MCs, separate cells from MCs, and dewater the harvested cells. The adherent cells on MCs were detached from MCs through enzymatic treatment and gentle mechanical force inside of mixer channels. Cells and MCs were then collected separately from spiral outlets and concentrated using the zig-zag concentrator unit. The dimension of the micromixer is shown in Additional file 1: Fig. S1

The spiral chip and zig-zag channel were produced as previously described (Razavi Bazaz et al. 2020a; Ding et al. 2022). Briefly, the devices were designed by SolidWorks and printed by the MiiCraft II 3D printer with a 10-µm layer thickness. Then the devices were rinsed with IPA and dried with an air nozzle three times. These devices were further post-processed by UV light in a UV-curing chamber and then bound to a PMMA sheet with a double-sided tape (ARclear^®^, Adhesive Research). Next, Tygon tubes (Tygon tubing, inner diameter: 0.50″, outer diameter: 0.90″) were used as connections of inlets and outlets to connect each part. Finally, the printed parts were then connected in series, as shown in Fig. 1.

### Characterisation of micromixer module

The performance of the micromixer has been evaluated using numerical (described in detail in Additional file 1: Sections S2, and S3 explained the detail of mixing index calculation) and experimental results. To verify the mixing efficiency of the mixers, food dye (1 mL in 49 mL DI water) and pure DI water were loaded in 50-mL syringes and injected into the mixer with syringe pumps at different flow rates. The syringes were connected to the Tygon tubes with precision syringe tips (0. 0. 50″ Long Tip, Adhesive Dispensing Ltd, UK). The pictures of the mixed liquid before and after going through the mixing units were taken by Olympus IX73 microscope (Olympus, Japan). The pictures were then analysed, and the degree of experimental mixing efficiency in these channels was compared with numerical results obtained using COMSOL Multiphysics (Razavi Bazaz et al. 2020b) (refer to Additional file 1).

### Characterisation of the microseparator module

Star-Plus MCs (Pall, SoloHill) were used to characterise the microseparator module. A spiral-shape microchannel was used for this purpose since it is capable of high-throughput and continuous sample processing without clogging issues (Moloudi et al. 2018). MCs were diluted in MACS buffer (Miltenyi Biotec, Australia) to acquire different volume fractions (0.1, 0.25 0.5, 0.75, 1% v/v%). The videos of particle movement were recorded by Phantom High-Speed camera (Phantom Academy, USA). The first 2000 frames of the video were stacked by ImageJ to observe the trajectory of the movement of the beads.

### Characterisation of the microconcentrator module

To concentrate the collected MSCs, a zig-zag microfluidic channel was designed and tested. Based on the spiral outlet dimensions, the input flow rates of the zig-zag channel were calculated (~ 1.6–1.8 mL/min). As such, 15 µm microbeads (PMMA (polymethyl methacrylate) latex beads, Magsphere, USA) were used to characterise different zig-zag channels with different dimensions. To this end, 50 µL microbeads were added into 10 mL MACS buffer and loaded into a 10-mL BD plastic syringe (BD, Australia). The microbeads were pumped through the device, and high-speed camera videos were recorded and evaluated.

### Microfluidic-based cell harvesting system setup

The modular 3D printed microfluidic system was set up with two micromixers, a spiral microfluidic device, and a zig-zag concentrator connecting in series with Tygon tubes. Cell harvesting was conducted in a biosafety cabinet to prevent any contamination. The upper mixer has two inlets, one was connected to the bioreactor through a peristaltic pump (Shenchen, China), and the other one was connected to a syringe pump (Fusion Touch, Chemyx Inc) for the TrypLE injection. Before cell harvesting, the whole setup was sterilised by 70% ethanol and UV irradiation. The same number of cell-attached MCs was harvested by the conventional membrane filtration method as a control. Briefly, the MCs were allowed to settle for 10 min, and then the culture media was carefully removed by a serological pipette. 40 mL of TrypLE was added to the bioreactor and incubated for 20 min. The microcarrier-cell suspension was gently pipetted before filtration. Lastly, the suspension was filtered by Steriflip Nylon Net filters (Millipore Steriflip filtration 100 μm, Merck, Australia), and the filtrated cells were collected. The recovery rate of cells and microcarriers were calculated by: $R={N}_{\mathrm{target outlet}}/({N}_{\mathrm{target outlet}}+{N}_{\mathrm{outher outlet}})$, *N* is the number of particles counted with haemocytometer. Counting was repeated 3 times.

### Cells culture and cells characterisation

Cells culture before harvesting and cells characterisation after harvesting are described in detail in Additional file 1: Section S4 and S5.

### Statistical analysis

The statistical significance in the data was calculated by Student’s *t*-test using Graph Pad Prism7 software. Significance levels were shown as *p < 0.05.

## Results

### Working principle of micromixer module

Two mixing strategies were applied in the proposed micromixer: Dean force induced by the helical 3D channel structure and the mismatch of flow rates induced by twisted helical groove structures following the 3D spiral. The first strategy creates fluid velocity mismatching in the channel’s inner side and outer side by having the curved channel, leading to the formation of two opposing vortexes in the channel and thus reducing the diffusion distance of the two fluids (Chai et al. 2021; Cai et al. 2017). For the second strategy, the twisted helical groove structure contributes to fluid mixing by creating a slow fluid flow zone and therefore inducing another mismatching of fluid velocity. This fluid mismatching carries the fluid from one side towards the other side of the channel, increasing the chance of fluids contact (Vasilescu et al. 2020; Chai et al. 2021); consequently, the increased contact of different fluids enhances the molecular diffusion. As previously reported, the groove designs in the channel would not introduce strong secondary flow (Tsui et al. 2008). Additional file 1: Fig. S3 shows the simulation results of the micromixer. Increasing the inlet fluid flow ratio leads to increased pressure in the system, which is negligible for smaller flow rate ratios and shows the system can be powered by normal lab-scale pumps. The cross-section 1 (CS1) across different flow rates in Additional file 1: Fig S3 shows that the chaotic advection phenomena dominate over diffusion when the flow rate increases. However, higher flow rate ratios do not necessitate a higher mixing index since fluids take time to mix and diffuse (Additional file 1: Fig. S3). Interestingly, velocity distribution for lower flow rates shows a symmetric profile along the channel length (Additional file 1: Fig. S3C), while it becomes asymmetric for higher flow rate ratios. This phenomenon might also contribute to the reduction of the mixing index at higher flow rates.

**Fig. 2:**
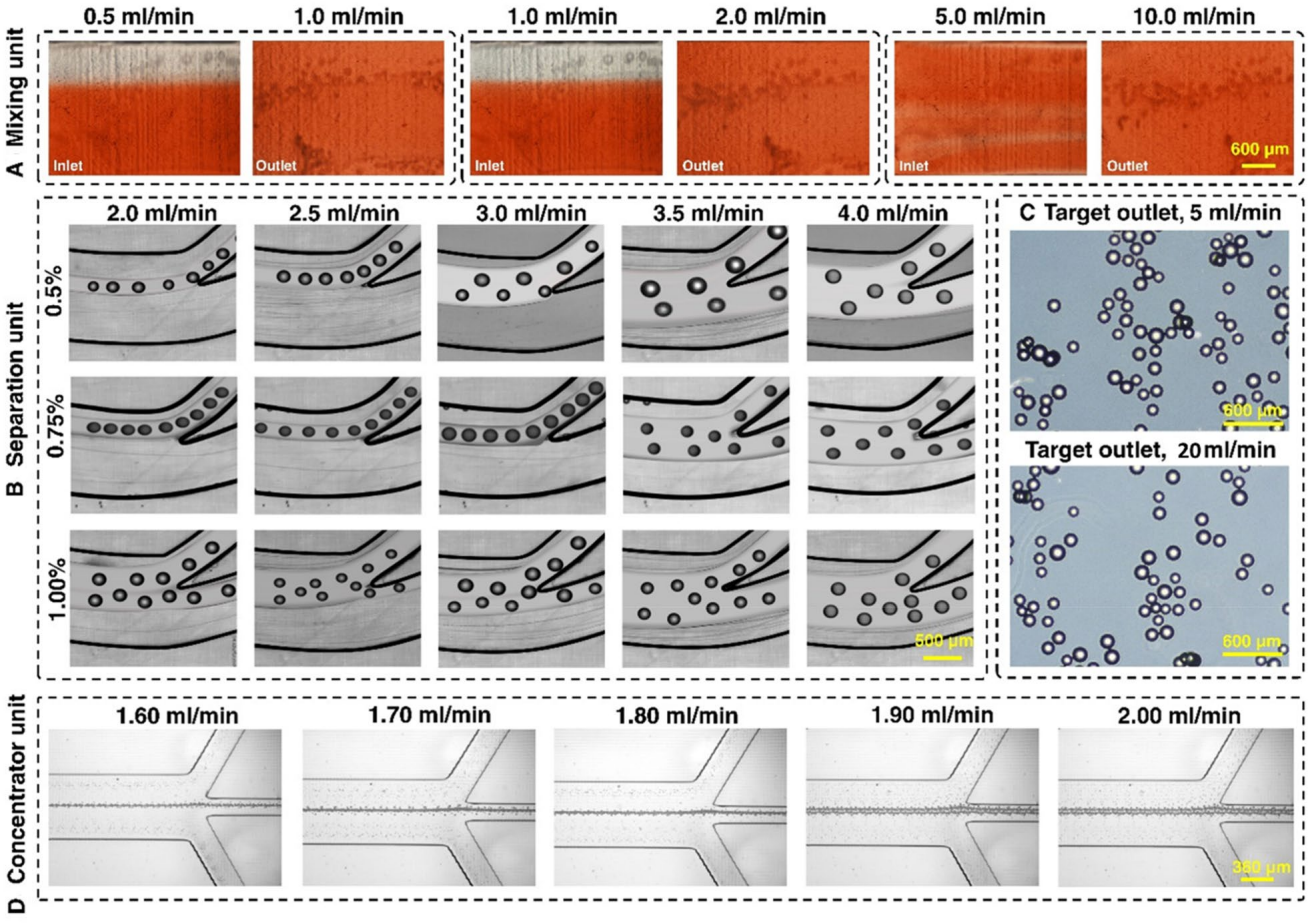
Characterisation of the microfluidic harvesting system using food dye, MCs, and fluorescent microparticles. **A** The micromixer reached 95% mixing efficiency with a 1:2 fluid flow mixing ratio. **B** The spiral microfluidic device can be operated at a flow rate of 3 mL/min with 0.75% v/v% microcarrier concentration. **C** The micromixers and spiral apply gentle forces to the microcarriers, and no breakage of microcarriers happened even when the flow rate was 6 times higher than the operation flow rate. **D** The zig-zag channel focuses 15 um beads from 1.6 to 2 mL/min with a 100% recovery rate

The experimental results of the mixing index with pure water and food dye for various flow rate ratios are illustrated in Fig. 2A. The mixing efficiency of the device was higher than 95% (Additional file 1: Fig. S5) at the flow rate ratio of 1 mL/min:2 mL/min. Hence, the total flow rate of 3 mL/min was chosen as an optimised flow rate for cell harvesting. Based on the method described in Additional file 1: Section S1, the experimental mixing index is 82.7%. The discrepancy between simulation and experimental results can be attributed to the difficulties of imaging 3D printed channels with microscopy and the addition of extra noise in the picture due to the unsmooth surface of the micromixer (Rouhi et al. 2021). The micromixers have no splitting, obstacles, or sharp turning, which are appropriate for processing cells without damaging them.

### Working principle of the microseparator module

The focusing position of microparticles inside a curved microfluidic channel is affected by two forces, inertial lift force (${F}_{\mathrm{L}}$) and Dean drag force (${F}_{\mathrm{D}})$ (Amini et al. 2014):

1 $${F}_{\mathrm{L}}=\rho \left(\frac{{U}_{\mathrm{max}}}{{D}_{\mathrm{h}}}\right){C}_{\mathrm{L}}{a}^{4},$$

2 $${F}_{\mathrm{D}}=5.4\times {10}^{-4}\pi \mu {De}^{1.63}a.$$

${F}_{\mathrm{L}}$ is affected by the density of fluid $\rho$, the hydraulic diameter ${D}_{\mathrm{h}}$ (which can be calculated by $4A/P$, $A=$ channel cross-section and $P=$ perimeter of the channel), the maximum fluid velocity ${U}_{\mathrm{max}}$ which is approximated as $2\times {U}_{\mathrm{f}}$ (${U}_{\mathrm{f}}$ is the average velocity), ${C}_{\mathrm{L}}$ which is a constant named dimensionless lift coefficient number and is dependent on the channel Reynolds number $(\mathrm{Re}=\rho {U}_{\mathrm{f}}{D}_{\mathrm{h}}/\mu , \mu$ is the viscosity of the liquid) and the diameter of particles $a$. ${F}_{\mathrm{L}}$ consists of two forces: shear-gradient and wall-induced lift force. Shear gradient lift force pushes the particles towards the wall due to the velocity difference between the middle area and the side area of the channel. When the particles move close to the wall, the wall lift force pushes the particles away. The balancing point of inertial equilibrium position contributed to the lift force is where these two forces balance each other (Razavi Bazaz et al. 2020c).

In a curved channel, the channel’s curvature causes the inner wall (IW) fluid to flow faster than the outer wall (OW) due to the shorter distance travelled. This transverse fluid flow creates another force that affects the focusing position of the particles, which is the Dean drag force (${F}_{\mathrm{D}}$). ${F}_{\mathrm{D}}$ is defined in Eq. (2), where $\mathrm{De}=\mathrm{Re}\sqrt{{D}_{\mathrm{h}}/2R}$ is the Dean number, and *R* is the radius of curvature; it describes the strength of ${F}_{\mathrm{D}}$. According to Eqs. (1) and (2), the forces applied to the particles are proportional to the particle size (${F}_{\mathrm{L}}\propto {a}^{4}, {F}_{\mathrm{D}}\propto a$). Therefore, different particle sizes have different focusing positions across the channel cross-section, and they can be collected through separate outlets (Mihandoust et al. 2020; Ozbey et al. 2019).

In a normal spiral channel, the particles inside the channel need to follow the rules of $\mathrm{Cr}>0.07$, where $\mathrm{Cr}=a/{D}_{\mathrm{h}}$ to be affected by the inertial forces inside the channel. In a scaled-up microfluidic channel, the increase in ${D}_{\mathrm{h}}$ results in a reduction of absolute flow velocity compared with a normal microfluidic channel. Therefore, the secondary forces applied to the microparticles were weaker, and the $\mathrm{Cr}$ value in the scaled-up microfluidic channel was much higher than the microfluidic channels $(\mathrm{Cr}>0.17)$ (Moloudi et al. 2019; Carlo 2009).

Another factor that affects particle focusing is channel rigidness. There is no swelling or channel inflation in rigid channels compared to traditional PDMS chips; thus, the scaled-up device should have theoretically a lower $\mathrm{Cr}$. Also, larger particles are more likely to be affected by mass and gravity since they are not neutrally buoyant (Moloudi et al. 2019), adding another variable despite flow velocity; the variable sizes of particles would also increase the difficulty in the channel design. When MCs and cells pass through the channels, focusing MCs near the IW causes the MSCs to be dispersed in the channel due to the large size difference between MCs and cells (MCs size are 150–220 µm, and MSCs are 15–20 µm). However, since large particles occupy the inner channel, the particle–particle interaction can stop some of the MSCs from going out through the inner outlet (Moloudi et al. 2018). Considering all these factors, in this study, we have designed the channel with a trapezoidal cross-section and heights of 550 µm and 620 µm, and a width of 1100 µm. This spiral chip has 6 loops and a slightly slanted enlarged inlet size to prevent clogging of MCs at the beginning of the channel (Fig. 1).

### Working principle of the microconcentrator module

The zig-zag channel relies on inertial, and Dean drag forces to focus the MSCs at the centre of the channel. When Reynolds number of the channel falls in the intermediate range 1 < Re < 100, the fluid flow is laminar, between Stokes and turbulent flow regimes. Therefore, inertial forces focus the randomly dispersed particles toward certain equilibrium positions after a sufficiently long channel length. As explained above, shear-gradient and wall-induced lift force are the main forces affecting the particle focusing in straight channels, and they both contribute to the overall inertial lift force ${F}_{\mathrm{L}}$. Straight channel relies on the difference in particle sizes to focus the particles at different positions (${F}_{\mathrm{L}}\propto {a}^{4}$). In zig-zag channels, Dean force ${F}_{\mathrm{D}}$ is introduced differently compared to the spiral microfluidic channel. The interchanging channel direction creates a mismatch of fluid flow velocity in an alternating pattern and introduces Dean force, accelerating the focusing of particles inside the channel.

A zig-zag channel has three focusing modes across different flow rates. When ${F}_{\mathrm{L}}{<F}_{\mathrm{D}}$, the particles focus at the side of the channels. When ${F}_{\mathrm{L}}{>F}_{\mathrm{D}}$, the particles were focused in the middle of the channel due to due to the strong ${F}_{\mathrm{L}}$. When ${F}_{\mathrm{L}}{\sim F}_{\mathrm{D}}$, particles are in the transition mode. For the aim of this study, MSCs need to satisfy the condition of ${F}_{\mathrm{L}}{>F}_{\mathrm{D}}.$ One primary advantage of the zig-zag channel is its operating ranges of flow rates, i.e., it can focus particles at the centre over a wide range of flow rates. After careful evaluations, the zig-zag channel with a cross-section of 360 µm × 60 µm, 60° angle has been proposed to concentrate cells after the spiral microfluidic device. To avoid clogging of zig-zag channels caused by the remaining MCs in the target outlet, some obstacles were planted at the target outlet of the spiral to ensure no MCs could enter the zig-zag channel.

### Pressure balance of microfluidic system

Combining multiple microfluidic devices in one system requires careful arrangement to balance the fluid flow and pressure change. An electronic circuit was used as an analogy for our system to understand better the fluid behaviour in the system (Additional file 1: Fig. S5). These microfluidic devices resemble the resistors that reduce the pressure input from the pumps, similar to the voltage drop in an electronic circuit (Oh et al. 2012). Keeping the flow rate and pressure stable according to the following equation is the key point of the successful operation of this system:

3 $$Q=\frac{\pi {{R}_{\mathrm{H}}}^{4}}{8\mu }\frac{\Delta p}{L},$$

where $Q$ is the volumetric flow rate, ${R}_{\mathrm{H}}$ is the hydraulic resistance of the channel, $\mu$ is the viscosity, $\Delta p$ is the pressure drop, and $L$ is the channel length. In a serial circuit, $Q$ (which is current *I* in the electronic circuit) remains constant in each device, thus ${Q}_{\mathrm{spiral}}={Q}_{\mathrm{mixer}1}={Q}_{\mathrm{mixer}2}$. ${Q}_{\mathrm{mixer}1}$ has two inputs, one from the peristaltic pump, and one from the syringe pump. In a parallel circuit, the current of the circuit ${Q}_{\mathrm{mixer}}={Q}_{\mathrm{inlet}1}+{Q}_{\mathrm{inlet}2}$. The working flow rates of micromixers and zig-zag channels are more flexible, while the spiral microfluidic device only works under a specific flow rate. To achieve this flow rate, we change the flow rate of the two pumps according to ${Q}_{\mathrm{spiral}}={Q}_{\mathrm{inlet}1}+{Q}_{\mathrm{inlet}2}$. The outlet’s resistance of the spirals affects the focusing of the MCs in the inner outlet. Therefore, the fluid pressure of the zig-zag channel must be balanced with the pressure-damping channel connecting to the inner outlet of the spiral device. This pressure-damping channel needs to have the same hydraulic resistance ${R}_{\mathrm{H}}$ to the zig-zag channel, which can be calculated by Eq. (4) (Oh et al. 2012):

4 $${R}_{\mathrm{H}}=\frac{8\eta L}{\pi {D}_{\mathrm{h}}},$$

where $\eta$ is the viscosity and $L$ is the finite length of the channel. Since ${D}_{\mathrm{h}}$ of the channel is fixed and ${R}_{\mathrm{H}}\propto 8L$, changing the length of the pressure-damping channel to reach R3 = R4 balances the pressure of the system and would not affect the particle focusing positions in the spiral channel (Additional file 1: Fig. S6). This system potentially eliminates the debris larger than cells through spiral channel, and removes debris smaller than the cells through the zig-zag channel.

### Evaluation of different modules with fluorescent microbeads and microcarriers

The maximum capacity and optimal flow rate of the spiral microfluidic device was determined by passing a different concentration of MCs through the device across a range of flow rate. As shown in Fig. 2B and Additional file 1: Fig. S6, from 2.0 to 4.0 mL/min, the focusing position of the MCs gradually shifts to the outer outlet. Noticeably, 3.0 mL/min is the critical flow rate that runs under high throughput while still focusing the MCs at the inner outlet. MCs with a concentration higher than 1% escape from the outer outlet even at a lower flow rate. However, MCs with a concentration of 0.75% can be sufficiently removed from the inner outlet at a flow rate of 3 mL/min. At the flow rate of 3 mL/min (2 mL/min from the bioreactor, 1 mL/min from the enzyme reservoir), the fluid mixing efficiency reached 95% after the first micromixer (Additional file 1: Fig. S4). The addition of the enzyme from the syringe pump inlet of the micromixer dilutes the sample.

The microcarrier concentration used for cell culture was 1.29% v/v% (1 g in 80 mL media). Therefore, MCs’ volume and concentration for cell harvesting before entering the microfluidic gadget were set to 70 mL to reach 0.75% when the sample arrived at the spiral microfluidic chip. The volume was calculated by the following equations: target concentration (0.75%)/dilution factor in micromixer (2/3)/concentration in culture (1.29%) × volume in culture (80 mL). As such, 40 mL of TrypLE was added since there was 30 mL of media inside the bioreactor after 50 mL of supernatant was taken away. The flow rate was set at 2 mL/min from the bioreactor and 1 mL/min TrypLE from the syringe pump, so the total flow rate of 3 mL/min fluid proceeded into the spiral. To demonstrate the inertial forces in the system do not damage the MCs, we passed MCs through the two micromixers and one spiral chip setup under a 20 mL/min flow rate. The results showed that the gentle forces applied by the micromixer do not change the shape and size of the MCs (Fig. 2C). Various inertial microfluidic channel designs can be used in this application as evidenced in our previous publications (Moloudi et al. 2018). In this study, we have showcased a rigid channel in the processing of large particle through the power of 3D printed inertial microfluidics.

The zig-zag channel was responsible for further concentrating the harvested cells. Since it was connected to the outer outlet of the spiral, the operation flow rate of the zig-zag channel needed to match the flow rate of the outer outlet of the spiral. The zig-zag concentrator was tested with 15 and 20 µm beads across different flow rates. The results showed that from 1.6 to 1.9 mL/min, the beads were concentrated 100% in the middle outlet (Fig. 2D). The beads were concentrated ~ 3.5 times, with ~ 70% of the volume removed, indicating good dewatering efficiency of the device.

### Application showcase

#### Harvesting MSCs from bioreactor using the microfluidic system

**Fig. 3:**
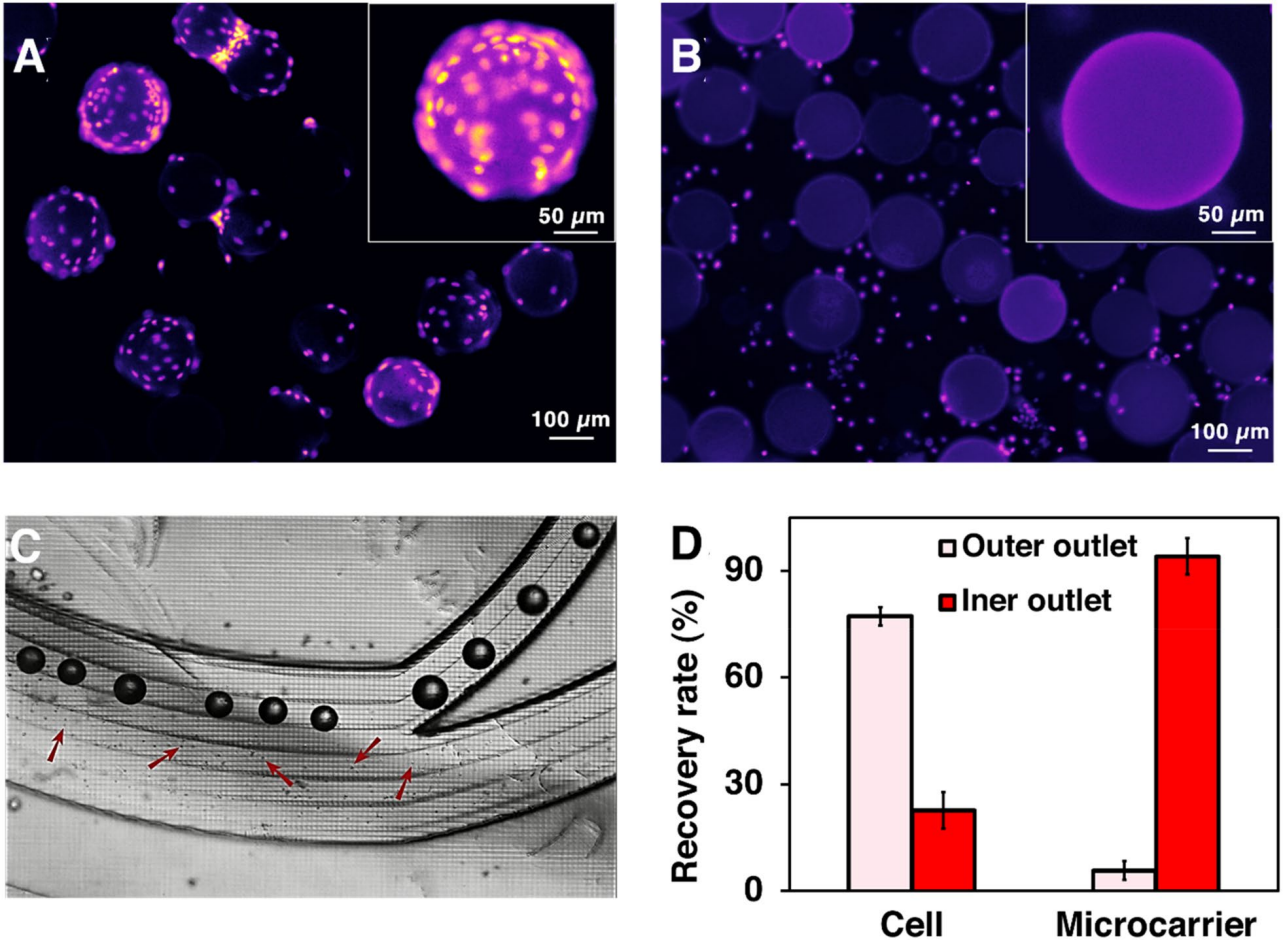
Harvesting MSCs with our microfluidic system. The concentrating efficiency of the zig-zag channel is shown in Additional file 1: Fig. S9. **A** and **B** Fluorescent microscopy images of cells–MCs before and after passing through the mixer. Cell nuclides were stained with Hoechst. **C** Separation of cells and microcarriers through the spiral chip. Cells and MCs were separated through different channels based on their size difference. **D** The recovery rate of cells and MCs after passing through the spiral chip in one round. The liquid collected from the inner outlet of the spiral was collected and performed a second-round running through to further recover the cells. The two-round separation results are shown in Additional file 1: Fig. S8

To investigate the efficiency of the microfluidic gadgets on cell detachment, the cells were stained with Hoechst before passing through the mixer. To ensure the complete detachment of cells in the micromixers, a one-inlet micromixer was added at the end to increase the interaction of cells and enzyme under the same mixing efficiency (Vasilescu et al. 2020). Figure 3A shows microcarrier-cell suspension before cell harvesting in which cells covered the whole surface of MCs. The growth of healthy MSCs on MCs commonly leads to cell–MCs aggregation (Ferrari et al. 2012) (Additional file 1: Fig S7). Therefore, to prevent the blockage of microfluidic devices, the cells–MCs suspension was incubated with enzyme for 5 min in the incubator to detach these aggregates. Figure 3B shows the MSCs were detached from MCs’ surface by enzymatic treatment and gentle mechanical force after passing through the micromixers.

The media containing detached cells and MCs from the micromixers were then passed through the spiral. Later, they were collected separately from two outlets (Fig. 3C). 94.11% of MCs were successfully removed in the first round of separation. 76.62 ± 2.1% and 17.21 ± 0.6% cells were recovered from the OW outlet in the first and second pass, respectively, and 6.16 ± 1.80% cell loss through the IW outlet at the end of the process (Fig. 3D, Additional file 1: Fig. S8). The sum of yield (sum of cells harvested from the OW outlet over the total cell harvest from all outlets) can reach ~ 94%. Adding some obstacles at the outlet leads to 100% of the microcarrier removal rate, making it ready for clinical applications. Additional file 1: Fig. S9 shows the tight focusing band of MSCs in the middle outlet and the removal of small debris in the outer outlets. The cell solutions were collected from the outer outlets, and no cell was found in the waste outlet. Cells were concentrated 4.5 times compared to the pre-filtered samples. Although the counting results showed that the recovery rate was higher than 100%, a small number of cell loss could potentially happen due to the heterogeneity, clumping of cells, or attachment to the tubing or channel walls.

#### MSCs viability and proliferation after microfluidic cell harvesting

**Fig. 4:**
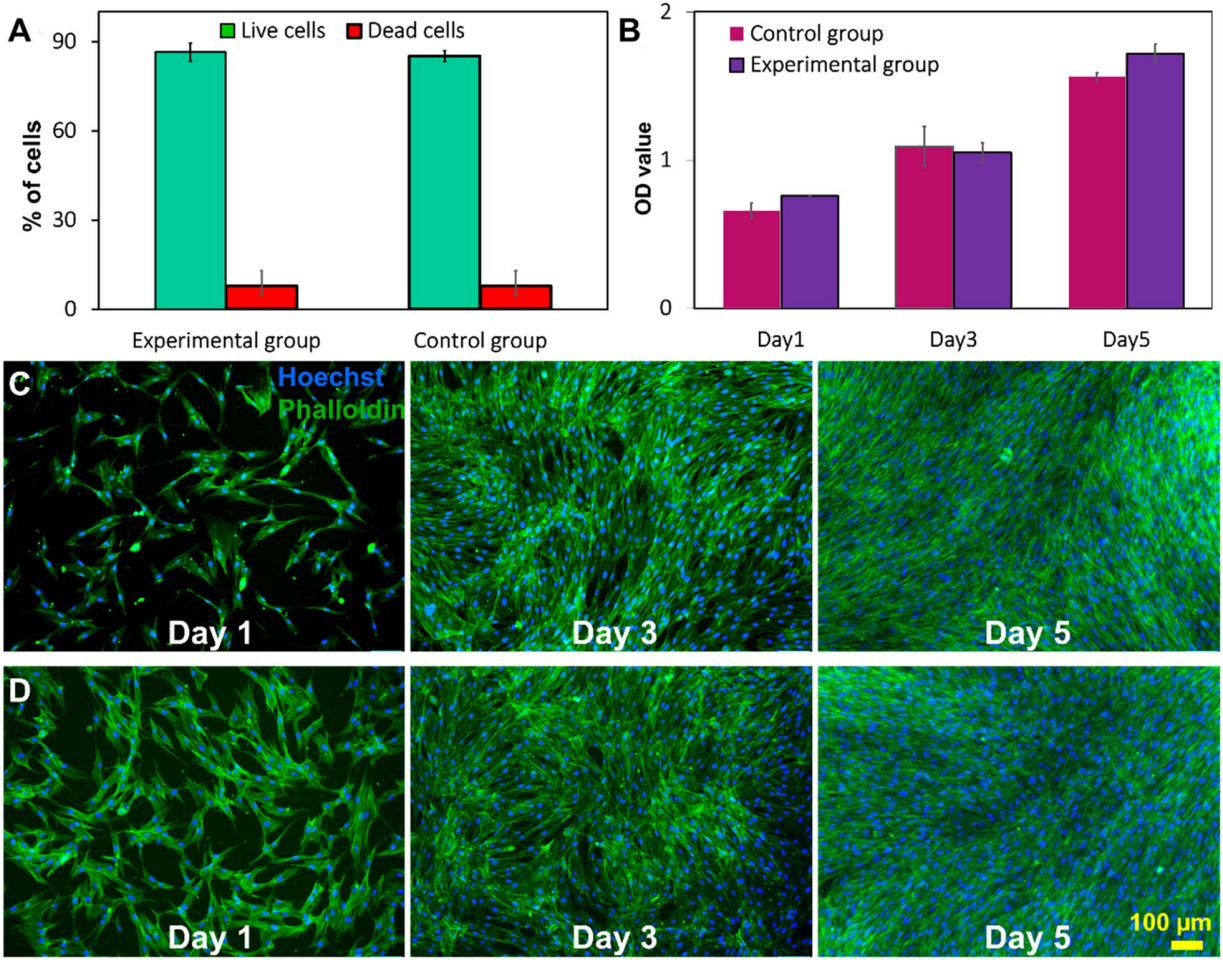
Viability and proliferation of MSCs after harvesting process. **A** The viability of cells harvested by the microfluidic system and filtration method. Cell viability did not change significantly compared with the control group. Data are presented as mean ± SEM (****p value < 0.0001, *n* ≥ 3). **B** MTS viability/proliferation rate of harvested cells. The morphology and proliferation rate of MSCs of the two groups were also compared with DAPI/phalloidin staining via **C** filtration group and **D** microfluidic group on 1st, 3rd, and 5th day of culture. F-actin filaments were visualised via FITC labelled phalloidin (green) and nuclei with DAPI (blue)

Cell viability was assessed immediately after harvesting. The live and dead staining results indicate that the microfluidic device did not compromise the viability of cells (Fig. 4A). MTS (3-(4,5-dimethylthiazol-2-yl)-5-(3-carboxymethoxyphenyl)-2-(4-sulfophenyl)-2H-tetrazolium) assay illustrating the metabolic activity of cells harvested by the device is also similar to the control. In the microfluidic group, the absorbance of media at 490 nm wavelength increased over time which indicates that cells have slightly higher metabolic activity than the control group, although the difference is not significant (Fig. 4B). Cell attachment, morphology, and proliferation were evaluated by staining the post-harvesting cells using DAPI and phalloidin. The fluorescent microscopy images in Fig. 4C and D indicate cells harvested with the microfluidic device have comparatively better cell attachment (Additional file 1: Fig. S10) than the control group on the first day of culture. After 3–5 days of culture, both groups of cells were confluent in the wells, and no significant difference in the growth rate was observed. Additionally, cells maintained their spindle morphology after harvesting with the device, and the size of cells was around 13–17 µm in both groups. The number of harvested cells after 1, 3, 5 days of cell seeding was counted by ImageJ to verify the MTS results. The results confirm that the microfluidic system does not affect cell attachment and growth after harvesting (Additional file 1: Fig. S10).

#### Stem cell properties and therapeutic properties of the harvested MSCs

**Fig. 5:**
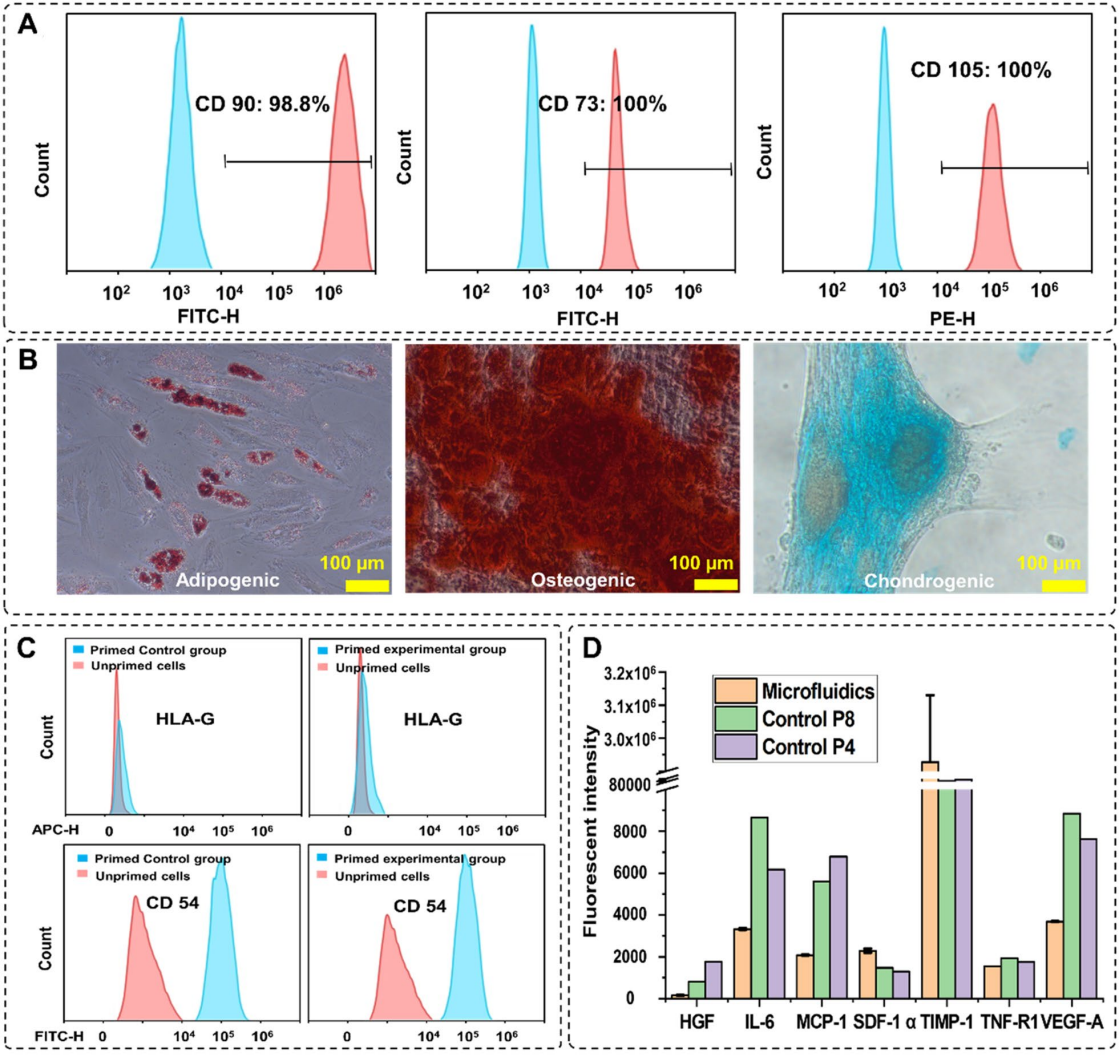
MSCs characterisation after harvesting. **A** Expression of the MSCs surface markers CD90, CD73, and CD105 after 3 passages of indicated cells preserve their stemness after harvesting. **B** Multipotency assay of harvested cells using Oil red (left), Alizarin red (middle), and Alcian blue (right) showed the cells maintained their capacity to differentiate into different cell types. **C** The expression level of the surface therapeutic proteins of the experimental group. The changes in the expression level of HLA-G and CD54 were similar in both groups. **D** Comparison of the cytokine secretion profile of MSCs harvested from the microcarriers with microfluidic system and the passage 4, passage 8 planar flask cultured cells

To confirm the stemness and multipotency of the harvested cells, the MSC surface markers were evaluated and trilineage differentiation was performed. CD90, CD73, and CD105 were stained with fluorescent antibodies (ThermoFisher, Australia) staining and counted by a flow cytometer (CytoFLEX LX, Beckman Coulter, USA). Figure 5A shows 98%, 100%, and 100% of the cells express CD90, CD73, and CD105, respectively, confirming the well-preserved MSCs identity. To assess the multipotency of cells after harvesting, cells were stained with Oil Red, Alizarin Red, and Alcian Blue staining after treating with adipogenic and osteogenic/chondrogenic induction media, respectively (Fig. 5B). Formation of bright red stain calcium deposits stained by Alizarin Red S confirmed osteoblastic phenotype of cells. Additionally, presence of red lipid droplets stained by Oil Red O verified the adipocyte phenotype, and the blue glycosaminoglycan complex staining showed the presents of chondrogenic cells. These results indicate that cells retained their differentiation potential.

The therapeutic effect of harvested MSCs is verified by staining the surface therapeutic proteins and analysis of the cytokines in the cultured supernatant. Figure 5C shows the changes in the expression level of the surface therapeutic proteins after priming with TNF-α and IFN-Υ for 24 h. HLA-G is a protein that prohibits the growth of lymphocytes, which expression level does not change with priming (Nasef et al. 2007; Selmani et al. 2009; Najar et al. 2012). The expression level of HLA-G in both microfluidic and control groups remained constant after priming. CD54 (iCAM) is a T-cell activation-related protein that is sensitive to inflammation, and the expression level of this protein increased significantly after priming (Rubtsov et al. 2017; Tang et al. 2018). Figure 5C shows that the expression level of CD54 increased 100% after priming in both groups. These results prove that there is no significant difference in the therapeutic properties of the MSCs after proceeding through the microfluidic device. Next, using the Custom ProcartaPlex Multiplex immunoassay panel, we analysed the secretion profile of the harvested cells compared to the secretion profile of cells passaged stably in multilayer cell factories. The results showed that the harvested cells expressed a similar or lower level of HGF, IL-6, CCL2, VEGF-A, and TNF-RI compared to the passage 4, passage 8 multilayer cell factory grown controls, the expression level of SDF-1 alpha and TIMP-1 are much higher than the control group (Fig. 5D).

### Multiplexing the microfluidic harvesting system for large-scale application

**Fig. 6:**
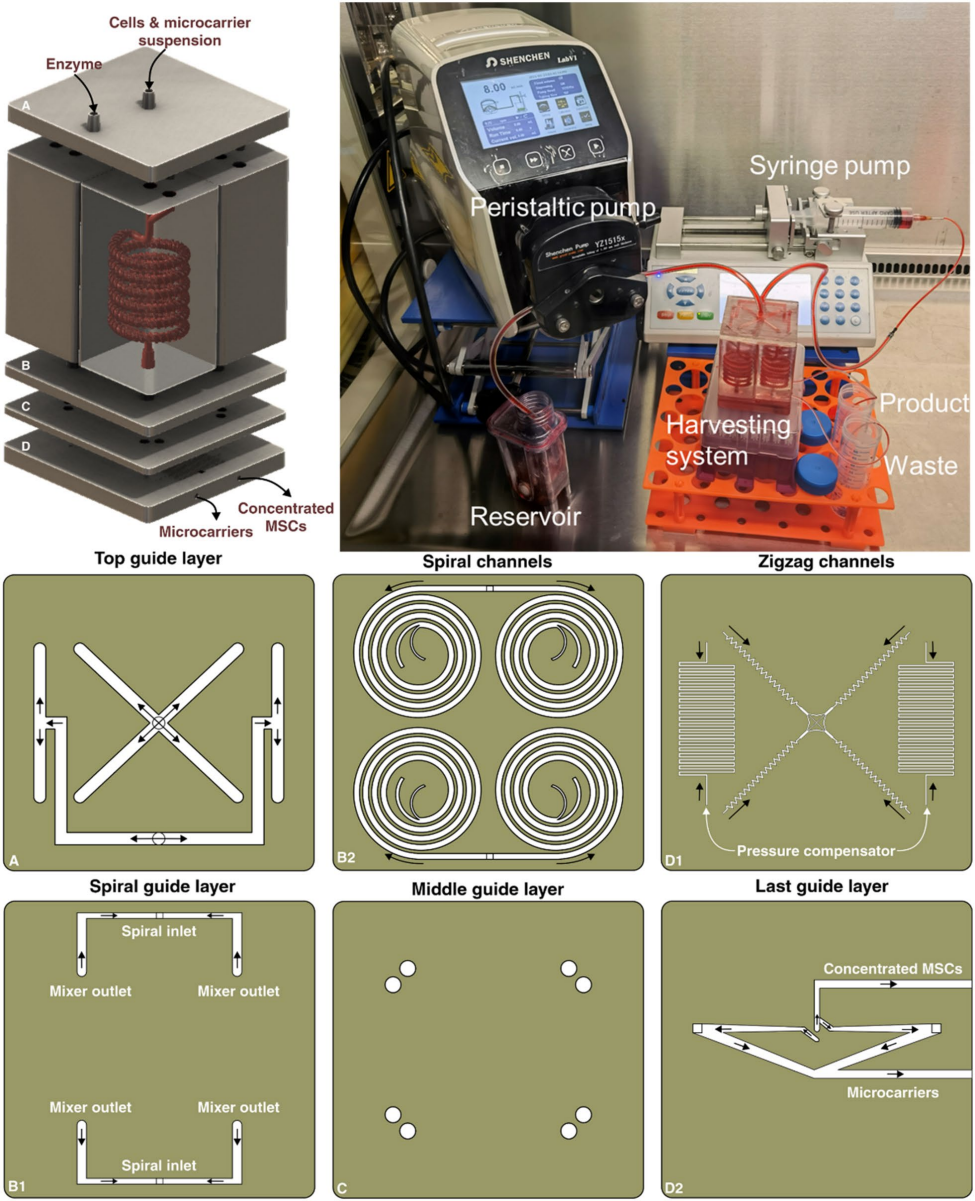
The setup and components of each layer. The multiplexing system consists of five layers: a top guide layer to distribute the liquid evenly into the micromixers; a micromixers layer that detaches MSCs from MCs; a spiral layer separating MSCs and MCs; a middle guide layer that provided a base for spiral and zig-zag channels and a zig-zag channel and pressure-damping channel layer that concentrate the MSCs. The cells and MCs are left from the outlets, respectively

A multiplexed system was built with the same printing protocols to demonstrate the capability of scaling up the microfluidic system for large-scale applications. The system consists of five layers (Fig. 6); the first layer is the fluid splitting layer; it has one inlet for cell and microcarrier solutions to enter the system and another inlet for the digestive enzyme with a flow rate of 8 mL/min for the cell and microcarrier solution and 4 mL/min for the enzyme. These two inlets split the total flow into four even sets and enter the 4 micromixers evenly in the second layer. The micromixers have inserted holes for the pins to anchor the positions and prevent leakage. The flow rate in each micromixer is 3 mL/min for detaching cells from MCs. The third layer is the spiral layer, with a pin inserted into the outlet of the micromixers. The solutions collected from each of the two micromixers were evenly split into two spiral microfluidic devices, and each spiral received 3 mL/min liquid flow to separate cells from MCs. Then, the fourth layer, a splitting layer was used as the bottom layer of the spiral. Two holes were opened at the outlets of the spirals, and this layer was bonded with the fifth layer spiral layer with double adhesive tape. Lastly, a whole 3D printed layer with 4 zig-zag channels and pressure-damping channels was attached to the splitting layer with double adhesive tape. The inner outlets of each spiral are connected to one pressure-damping channel, and the outer outlets of each spiral are connected to one zig-zag channel. The cross-sectional area ratio of the inner and outer outlet is 2:3; the flow rate of the outer outlet is, therefore 1.8 mL/min for each spiral. As shown in Fig. 2, the zig-zag channel can focus the cells from 1.4–1.9 mL/min. This flexible working range of the zig-zag channel ensures the cells focus on the middle outlet of the device and reduce the requirement of precision of the pressure-damping channel. The total flow rate of the cell outlet was 7.2 mL/min, while the MCs outlet was 4.8 mL/min.

## Discussion

The merits of microfluidic devices, such as low-cost, high throughput, labour-free, customisability, and energy efficiency, meet the need of the bioprocessing industry. Recently, multiple attempts have been made to bring microfluidic devices to solve the challenges associated with bioprocessing. However, microfluidic devices are still facing difficulty in accommodating and integrating themselves in the bioprocessing industry. In this manner, 3D printing technology can be used as a bridge to connect microfluidic devices and the bioprocessing industry. The one-step fabrication method of 3D printing technology (printing and washing) allowed us to test 16 zig-zag channels with different dimensions, six different inertial concentrator designs and three micromixers.

In our proposed microfluidic system, cells detachment, separation, and concentration–time are short, 5 min for incubation and 20 s for passing through the system with a total length of < 5 cm. This short processing time could effectively minimise the negative impact of enzymatic treatment on the cell membrane and enhance attachment and growth of harvested cells (Fig. 4), indicating well-preserved cell membrane integrity and functionality. Although the damages caused by enzymatic treatment can be reversible (Tsuji et al. 2017), it takes a few passages for the cells to recover and is not feasible for clinical applications.

The results of cell viability and MTS assays indicate that the viability and proliferation rate of the microfluidic-harvested cells are the same as the control. This is in agreement with the results reported by Nienow et al. (2016), who suggested that agitating cell–MCs suspension facilitates cell detachment while not compromising cells’ properties and viability. As expected, the cells maintain their differentiation potential trilineage (Fig. 5D), their size, spindle morphology (Fig. 4D), and surface markers expression (Fig. 5A). The size and morphology of the cells are important indicators of the cell potencies and secretion profile since different sizes MSCs were shown to have a different expression levels of differentiation promotor/inhibitor genes and different secretion levels of therapeutic factors (Yin et al. 2018, 2020; Lee et al. 2014).

Our experiment showed that the anti-inflammatory surface proteins expression level of the harvested cells during the subsequent subculture had no difference compared to the control group (Fig. 5C). This indicates that the cells preserved their therapeutic properties after the process, and the microfluidic system is safe for the industrial production of stem cells for clinical purposes. The high secretion level of SDF-1α and TIMP-1 proteins suggest strong potential in therapeutic applications. However, these results are not enough to draw the conclusion of whether this harvesting method alters cytokine secretion levels of the MSCs. Previous works show that the topography of the culture system (Leuning et al. 2018) and shifting from 2 to 3D culture (Russell et al. 2018) influenced the cytokine expression level of cells. Ng and Wang (2021) showed that even growing cells on different types of microcarriers influence the secretion profile. Therefore, the secretion profile changes caused by our 3D printed modular harvesting system require further characterisation. These results showed that the cells harvested with our 3D printed modular microfluidic system preserved all the cell properties with no cytotoxic effect, and damage caused by the material, or the hydrodynamic forces was observed.

With the aid of 3D printed technologies (Additional file 1: Section S6), our microfluidics system has multiple advantages over the current laboratory and industrial adherent cell harvesting methods. This microfluidic system requires only two pumps to trigger, and no complicated tubing and valve is needed. This system is cGMP compatible and the design of the system ensures negligible risk of contamination (Tamura et al. 2012; Caruso et al. 2014); The device can be operated in a continuous manner, which is particularly suitable for industrial-scale application (Castilho and Medronho 2002); The system can be used as a single unit system for lab-scale production or easily scaled-up by paralleling the devices together for large volume processing; Other microfluidic devices can also be integrated to perform other functions such as quality control of cellular products (Ding et al. 2021). With the small device footprint, reaching 2 L/min flow rate requires 100 chips, and the total volume would be only 1 m^3^. It will take 25 min to harvest 50 L MCs. The small footprint allows easy integration into any current-available system, 3D printing technologies allow easy and rapid prototyping of customised fluidic interconnects at a low cost to aid the industrial integration (Ho et al. 2015).

On the other hand, our system shows clear advantages over TFF (Schnitzler et al. 2016) with its clogging-free operation manner. This important feature reduces the production cost since the device does not need frequent membrane replacement and maintenance and can be single used due to the low-cost. Also, the low flow rate in each individual unit of our device ensures the cells are not suffering from shear stress like TFF, resulting in cell damage (Cunha et al. 2015). Moreover, this system can be integrated into other enzymatic detachment methods or even enzyme-free cell detachment procedures as well. In recent years, frontier research about smart MCs shows that thermosensitive MCs and soluble MCs have great potential in future cell culture (Tamura et al. 2012; Kalra et al. 2019; Hanga et al. 2021). Proceeding these MCs through our microfluidic gadget may increase exposure to light and heat while benefiting from the agitation of fluid flow. In our multiplexing design, we showcased the first multiplexed modular microfluidic system. The system is built in a nonlinear and modular manner which has not been showcased before. This rapid, low-cost prototyping is not possible without 3D printing technology.

## Conclusion

In this paper, we proposed a 3D printed modular microfluidic system consisting of three modules, which are micromixer, microseparator, and microconcentrator, to detach and separate MSCs from MCs. Each module was produced with direct SLA printing, creating highly accurate 3D structures with a low cost and a simple, rapid manufacturing process. Operating at the throughput of 3 mL/min, this microfluidic gadget can detach the cells fully from MCs with 5 min incubation time and 20 s proceeding time through the device, removing 100% of the MCs from cells solution while recovering 77% of cells in one round. The cells passing through the device were viable proliferative with preserving their differentiation potential. More importantly, the therapeutic potential of the cells was well preserved. Our scaled-up version shows that the current system has the potential to apply in the stem cell industry in cGMP compatible manner. Compared to the current system, this gadget is operated in high throughput and clogging-free manner. It simplifies the cell harvesting procedure, minimises the damage and chance of contamination to the cells, and reduces the overall production cost on a large scale. Furthermore, this system is flexible and can potentially be modified to fit with any microcarrier and bioreactor to produce various cell types and products.

### Supplementary Information

**Additional file 1.** The design criteria, methodology and simulation sections of the microfluidic system, methodology of cell culture and properties measurement, supplementary figures of the microfluidic system characterisation and MSCs characterisation post-harvesting.

## Data Availability

All data that support the findings of this study are included within the article and Additional file 1.

## Abbreviations

MSCs: Mesenchymal stem cells
MCs: Microcarriers
TFF: Tangential flow filtration
cGMP: Current good manufacturing practice
MTS assay: 3-(4,5-Dimethylthiazol-2-yl)-5-(3-carboxymethoxyphenyl)-2-(4-sulfophenyl)-2H-tetrazolium Assay
FDM: Fused Deposition Modelling
SLA: Stereolithography
DLP: Digital light processing
2PP: Two-photon polymerisation
IW: Inner wall
OW: Outer wall
