# Supplementary material for: A modular 3D printed microfluidic system: a potential solution for continuous cell harvesting in large-scale bioprocessing
Source: Bioresour Bioprocess. 2022 Jun 6;9(1):64. doi: 10.1186/s40643-022-00550-2 (PMC10991575; doi:10.1186/s40643-022-00550-2)
Supplement: Supplementary file 1 — Additional file 1. The design criteria, methodology and simulation sections of the microfluidic system, methodology of cell culture and properties measurement, supplementary figures of the microfluidic system characterisation and MSCs characterisation post-harvesting. [file 40643_2022_550_MOESM1_ESM.docx]

**A modular 3D printed microfluidic system: a potential solution for continuous cell harvesting in large-scale bioprocessing**

Lin Ding^1,^ ^Ѱ^ Sajad Razavi Bazaz^1^ ^Ѱ^, Mahsa Asadniaye Fardjahromi^1,2Ѱ^, Flyn McKinnirey^3­^, Brian Saputro^3^, Balarka Banarjee^3^, Graham Vesey^3^, Majid Ebrahimi Warkiani^1,4*^

^1^School of Biomedical Engineering, University of Technology Sydney, NSW, 2007, Australia

^2^School of Engineering, Macquarie University, Sydney NSW, 2109, Australia

^3^Regeneus Pty Ltd, Paddington, Sydney, NSW, 2021, Australia

^4^Institute of Molecular Medicine, Sechenov University, Moscow, 119991, Russia

^Ѱ^ These authors contribute equally

*Contact

Majid Ebrahimi Warkiani ([majid.warkiani@uts.edu.au](mailto:majid.warkiani@uts.edu.au))

School of Biomedical Engineering, University Technology Sydney, Sydney, New South Wales 2007, Australia

**Supplementary information section**

**S1. Microfluidic system design consideration**

Based on previous developments of scaled-up microfluidic devices [1-3], here, we used a 3D printed scaled-up modular microfluidic system consists of two micromixers, one spiral inertial separator, and one zig-zag concentrator to detach and separate cells from microcarriers (MCs) and dewater the cells. The micromixers in this system assisted cell harvesting by mixing the MCs and enzyme sufficiently and inducing mild mechanical force, reducing the cell detachment time. 3D passive micromixer has been utilised in this system owing to its high mixing efficiency, adaptable flow rate range, adequate cross-sectional channel size, and gentle forces applied to the cells. MCs were found to be fragile and have the risk of breakage in stirring bioreactor [4], but they remained intact even under high flow rate in our micromixers (Fig. 2C), and no clogging of channels was observed [5-7]. For the same reasons, spiral microfluidics was chosen due to the large channel dimension and gentle forces it applied to the microparticles and in the last step, the zig-zag channel was used to concentrate the cells due to its high dewatering efficiency and flexible working flow rate range. In addition, the spiral channel extracts larger particles out of the cell solution, and zig-zag channel removes smaller debris like microplastics of the culture flask, cell debris or MCs fragments (Fig. 2) which might trigger immune rejection [8] of the patients. These two devices worked together to remove particles and debris that are bigger or smaller than the cells.

**S2. Numerical simulation of the micromixer**

To characterise the performance of the micromixer, the fluid behaviour in the micromixer was simulated using COMSOL Multiphysics 5.5 [5]. Two equations, Navier-Stokes, and continuity equations (Eqs. (1) and (2)), which are regarded as the governing equations for laminar, incompressible Newtonian fluid, were used to calculated velocity and pressure distribution in the channel:

| $\nabla.V=0$ | (1) |
| --- | --- |
| $\frac{\partial V}{\partial t}+\rho\left( V.\nabla\right)V=-\nabla P+\mu\nabla^{2}V$ | (2) |

where $V$ is the velocity vector (m/s), $\rho$ is the fluid density (kg/m^3^), $\mu$ is the dynamic viscosity (N·s/m^2^), and $P$ is the pressure (Pa). The fluid velocity and pressure in Eq. (2) can then be used to model the mass transport of the fluid by the convection-diffusion equation (Eq. (3))

| $\frac{\partial c}{\partial t}+\left( V.\nabla\right)c=D\nabla^{2}c$ | (3) |
| --- | --- |

where c is the fluid concentration (Mol/m^3^), D is the diffusion coefficient (m^2^/s).

To evaluate the performance of the micromixer, mixing index was introduced:

| $MI=1-\sqrt{\frac{\frac{1}{N}\sum_{i=1}^{N} {{(c}_{i}-\bar{c})}^{2}}{{\sigma^{2}}_{max}}}$ | (4) |
| --- | --- |
| ${\sigma^{2}}_{max}=\bar{c}(1-\bar{c})$ | (5) |

where N is the total number of the cross-section mesh elements, ${\sigma^{2}}_{max}$is the maximum variance of mixture concentrations at the specific cross-section along the channel, $c_{i}$ is the concentration of species for each of the mesh elements, and $\bar{c}$ is the average value of $c_{i}$. The value of $\bar{c}$ is related to the inlet volume ratio, which is 2:1 in our case, and it is equal to 0.66 [9].

**S3. Calculation of experimental mixing index**

To extract the experimental values of the mixing index, an image processing software (Fiji, NIH, USA) has been used. Grayscale values of the images along a line parallel to the channel width at the inlet and outlet were extracted from the pictures. These values were then normalized using Eq. (6).

| $I_{i}=\frac{I_{i}^{*}-I_{min}^{*}}{I_{max}^{*}-I_{min}^{*}}$ | (6) |
| --- | --- |

where the actual intensity is represented by $I_{i}^{*}$, and the minimum and maximum values of intensity at the inlet (unmixed fluid) are given by $I_{min}^{*}$ and $I_{max}^{*}$, respectively. The experimental value of the mixing index is then calculated using Eq. (9).

| ${MI}_{experimental}=1-\frac{ME}{{ME}_{no mixing}}$ | (7) |
| --- | --- |

Here, $ME$ and ${ME}_{no mixing}$ were calculated using Eqs. (10) and (11).

| $ME=\sqrt{\frac{1}{N}\sum_{i=1}^{N} \left( \frac{I_{i}-\bar{I}}{\bar{I}} \right)^{2}}$ | (8) |
| --- | --- |
| ${ME}_{no mixing}=\sqrt{\frac{1}{N}\sum_{i=1}^{N} \left( \frac{I_{min,max}-\bar{I}}{\bar{I}} \right)^{2}}$ | (9) |

where $N$ is the number of pixels in the inlet or outlet image along a line parallel to the channel width, $\bar{I}$ is 0.5, and $I_{min,max}$ can be 0 or 1, respectively.

**S4. Cell culture in culture flask and bioreactor**

Human adipose-derived mesenchymal stem cells were supplied by Regeneus Ltd. The cells used in this study were in passage 8. The cryopreserved cells were thawed and cultured in complete media made of α-MEM (Gibco, Australia) supplemented with 10% Human Platelet Lysate (HPL, COOK Regentec, US) in a 37^o^C incubator with 5% CO_2_ before seeding on microcarriers.

Star-Plus MCs were sterilised by autoclaving according to the manufacturer's instruction and then were suspended with a density of 12.5 mg/ml prior to cell seeding. MCs were transferred to the 100 ml PBS mini bioreactor (PBS biotech, USA), and the cells were seeded at a concentration of 2.0 × 10^6^ cells/g MCs in the bioreactor. The bioreactor was placed at 37 ^o^C humidified bioreactor with 5% CO_2_. To improve cell attachment, the bioreactor was first operated at 15 rpm for the first 24 hours and then increased to 24 rpm to attain near-full suspension of MCs-cells suspension. Cell propagation on MCs proceeded for 7 days. After that, cells were harvested by the traditional filtration method and the proposed 3D printed integrated microfluidic system. To visualise the number of attached cells on the MCs after cell harvesting, the cells were stained with Hoechst staining (Miltenyi Biotec, Australia) and observed by fluorescent microscope (Olympus IX73, Olympus, Japan). Briefly, 1 mL of the cultured microcarrier-cell suspension was taken out from the bioreactor, rinsed with PBS, and then fixed by 4% paraformaldehyde (Sigma Aldrich, Australia) for 20 min. Then the cells-attached MCs were washed with PBS and stained with Hoechst for 30 min.

**S5. Cells characterisation after passing through microfluidic devices**

## S5.1 Cell viability assay and proliferation assay

The viability of cells after harvesting was evaluated by live and dead cells viability assay kit (ab115347, Abcam, UK) and viability profile for control and experimental group was assessed via flow cytometry (CytoFLEX LX, Beckman Coulter, USA) according to the manufacturer's instructions. The proliferation rate of harvested cells was quantified by MTS. To this aim, 2× 10^4^ cells/well were seeded in three wells of the 24 well plates, and after 1, 3, and 5 days of cell seeding, the proliferation rate of cells was examined by MTS reagent containing serum-free medium according to the manufacturer's protocol (Progmo, Australia). The absorbance was measured at 450 nm using a Tecan Spark multimode microplate reader (Tecan, Switzerland).

To further quantified cell proliferation, the cells (2 × 10^4^ cells/well) were seeded on a 24-well plate and on the 1^st^, 3^rd,^ and 5^th^ day of culture, cell nuclei and F-actin filaments were stained with DAPI and FITC-labelled phalloidin, respectively. Cells were visualised using Olympus IX73 microscope (three fields of view per replicate; three replicates; Olympus, Japan). Then the number of cells for each sample was counted manually using ImageJ.

## S5.2 Identification of cell phenotype

To evaluate the microfluidic harvesting system's long-term effect on the stem cell, the surface markers of MSCs were stained by fluorescent antibodies. 3×10^4^ cells were seeded in 24-well plates and incubated at 37^o^C and 5% CO2. When cells reached near 80% confluence, they were labelled with monoclonal antibodies CD90-FITC, CD105-PE, and CD73-FITC (Abcam, Australia), fixed by 4% formaldehyde (Sigma Aldrich, Australia) and analysed by CytoFLEX flow cytometer and FlowJo software (BD, USA).

**S5.3 Trilineage differentiation**

The harvested cells were seeded in 24-well plates with a density of 3×10^4^ cells/well. The detail method was reported in our previous work [10]. Briefly, three days after culture, the media was replaced with StemPro™ Osteogenesis differentiation media (Gibco, Australia) for osteogenic and differentiation, StemPro™ Chondrogenesis differentiation media (Gibco, Australia) for chondrogenic and StemPro™ adipogenic differentiation media (Gibco, Australia) for adipogenic differentiation. After 21 days, the osteoblasts property of cells was verified by Alizarin Red staining (Sigma Aldrich, Australia). The cells first were fixed with 70% ethanol for 1 hour, washed with deionized water several times, then stained with alizarin red s solution, and incubated at room temperature for 1 hour. The cells treated with chondrogenic and adipogenic differentiation media were stained with Alcian Blue and Oil Red O (Sigma Aldrich, Australia) staining respectively. Both group of cells were fixed with 4% formaldehyde for 1 hour, and then Oil Red staining was added into the adipocytes for 30 minutes staining and Alcian Blue was added into the chondrocytes and stain the cells overnight. Finally, the cells were washed with deionized water three times, and the stained matrix was observed by a light microscope (Olympus, Japan).

## S5.4 Surface proteins responses to cytokine stimulation

The harvested cells were seeded into a 6 well plate at the concentration of 5×10^4^ cells/ml in complete media. After cells reached 50% confluence, the cells were treated with priming media containing α-MEM with 5% HPL, 10ng/mL TNF-α, and 100ng/mL IFN-γ recombinant proteins (Stem cell technologies, USA). The unprimed cells (treated with α-MEM and 5 % HPL) were used as a control. After 24 hours, cells were harvested and centrifuged at 800 G for 7 minutes. The supernatant was discarded, and the cell pellet was resuspended in 1mL of cold DPBS (2-8 ℃) and stained with CD54 (iCAM) and HLA-G antibodies (Biolegend, Australia). The labelled cells were analysed via a CytoFLEX LX flow cytometer system and CytExpert software.

## S5.5 Cytokine secretion study

To assess the characteristics of MSCs secreted protein, MSCs were assessed under normal culture conditions and after harvesting from the microfluidics system. Analytes, including HGF, IL-6, MCP-1, VEGF-A, SDF-1α, TNFR1, and TIMP-1 were measured using the Custom ProcartaPlex Multiplex immunoassay (Thermo Scientific, Australia). ProcartaPlex is a Luminex-based multiplex assay that utilizes magnetic beads with antibodies directed against distinct analytes in one assay, enabling the measurement of many analytes at one time. Assays were performed as per the manufactures' instructions. Briefly, MSCs were defrosted and spun at 10,000g for 2 minutes; spun sample supernatant is bound with detection antibodies added, conjugated with Streptavidin-PE, and the fluorescent intensity of each sample was read on a MAGPIX 200 (Luminex Corporation, US) reader.

**S6. Additive manufacturing for device fabrication**

In our system, all microfluidic devices were fabricated by 3D printing. 3D printing is the only choice to fabricate such spatial designs in a short time due to the high customisability, high accuracy, and potentially labour-free manufacturing process. Stacking of PDMS [11], laser cutting [12] or micromilling [13] have limited capability of fabricating 3D structures, and they are expensive and labour-intensive. An alternative fabrication method is direct 3D printing of microfluidic devices, which have great potential in the industry due to their rigid nature, highly customisable design, cost-effective, and rapid manufacturing process [14-16]. These devices required manual aligning and binding and are therefore prone to human error. Micromixers with such complex geometry can only be made using sacrificial moulds or direct printing, similar to the one that we used in previous study [17]. However, sacrificial moulds do not have high quality (limited on the accuracy of the printer) and hugely rely on experienced users to produce, it has an extended processing time, and excessive PDMS material is needed. Therefore, in this paper, we employed the SLA 3D printer to directly print the micromixer, shortening the cost, time, and effort needed to fabricate the device with the same spatial and precise geometry. With this printing procedure, the micromixer and spiral chip can be fabricated within a few hours and require only very simple post-processing steps, which can be automatically proceeded by washing and curing machine to further reduce labour cost.


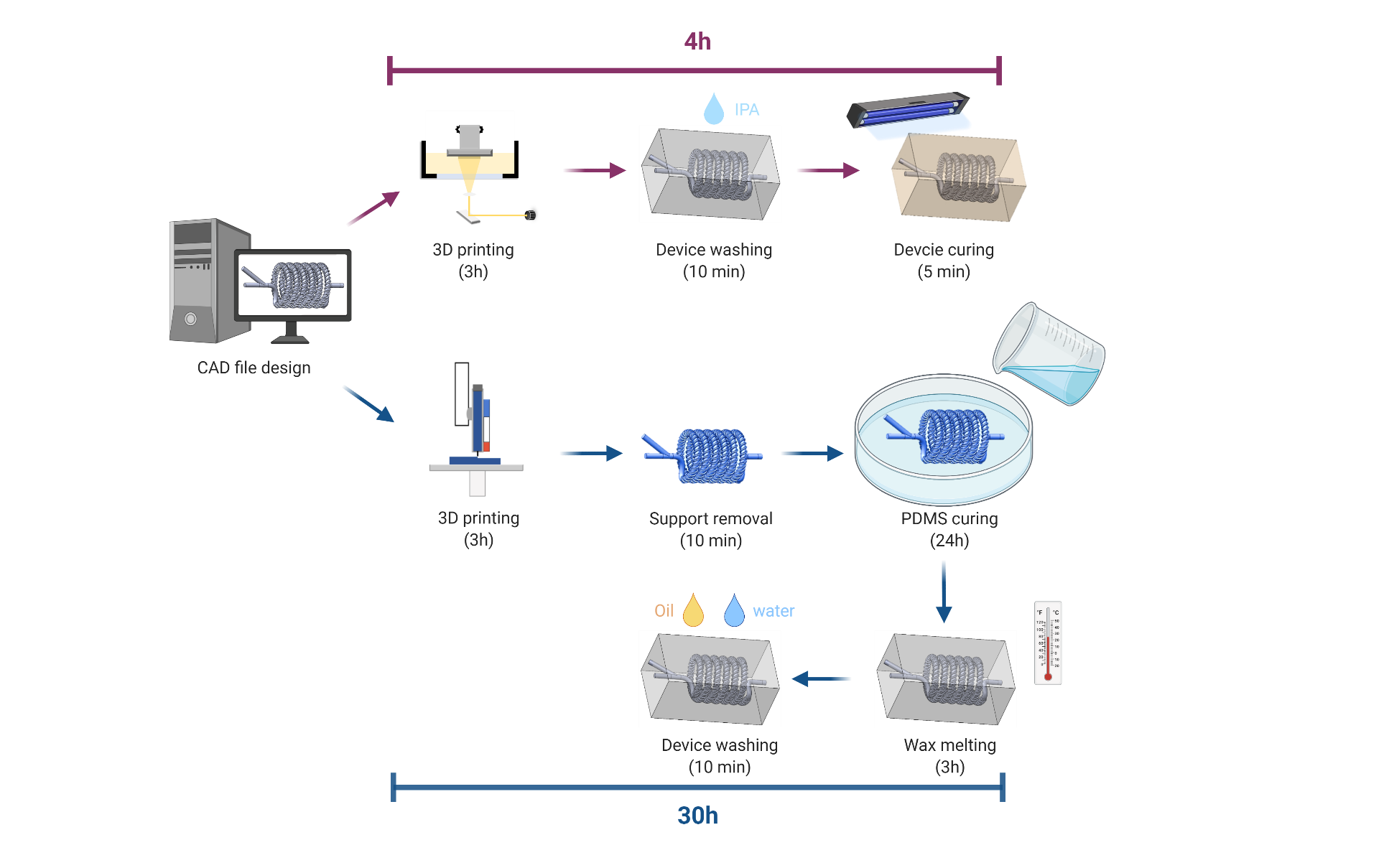


**Fig. S1.** Direct printing of 3D micromixers hugely reduces the time needed for fabricating time compared to wax printing method.


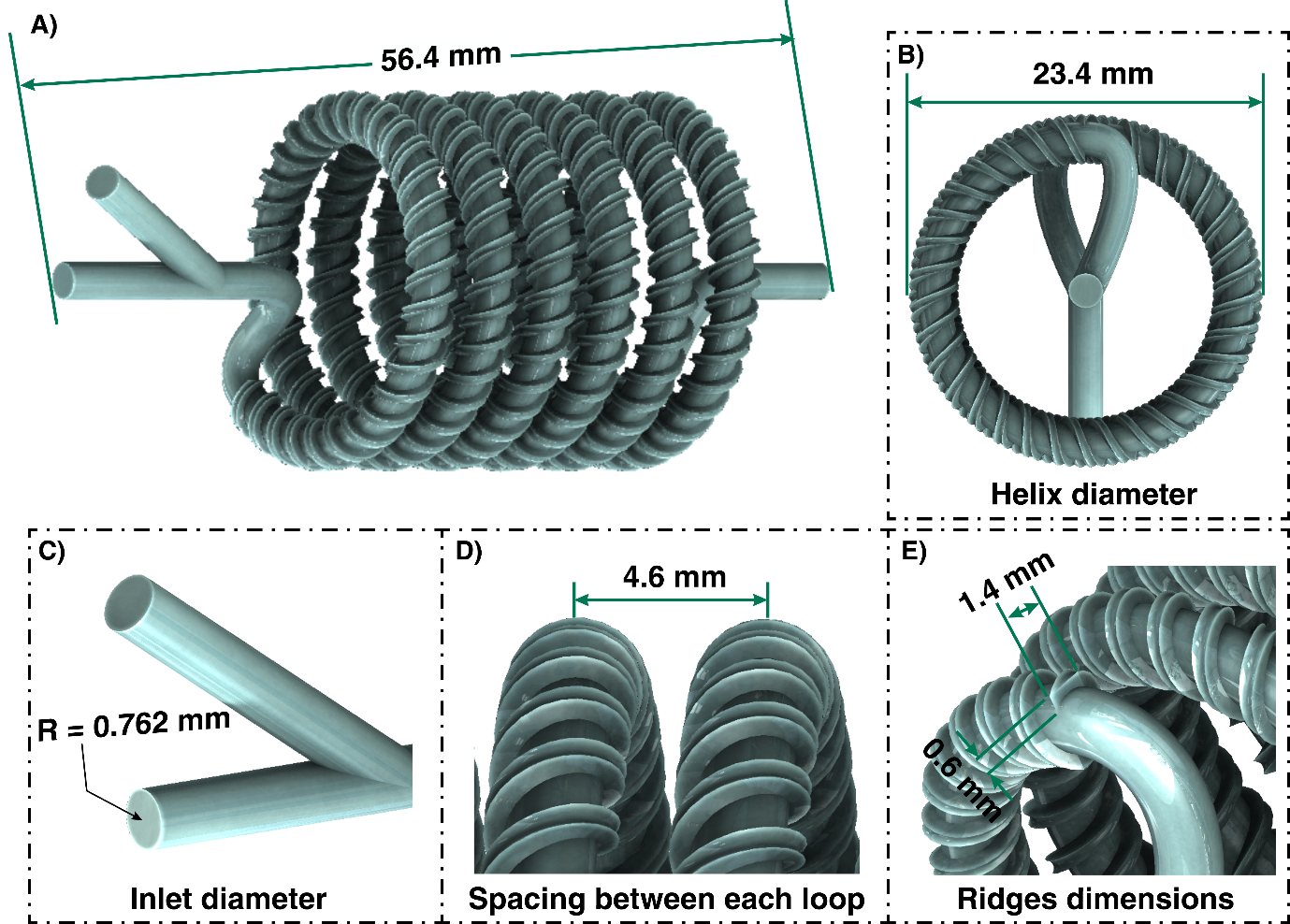


**Fig. S2.** Dimensions of threaded micromixer used for detachment of cells from microcarriers


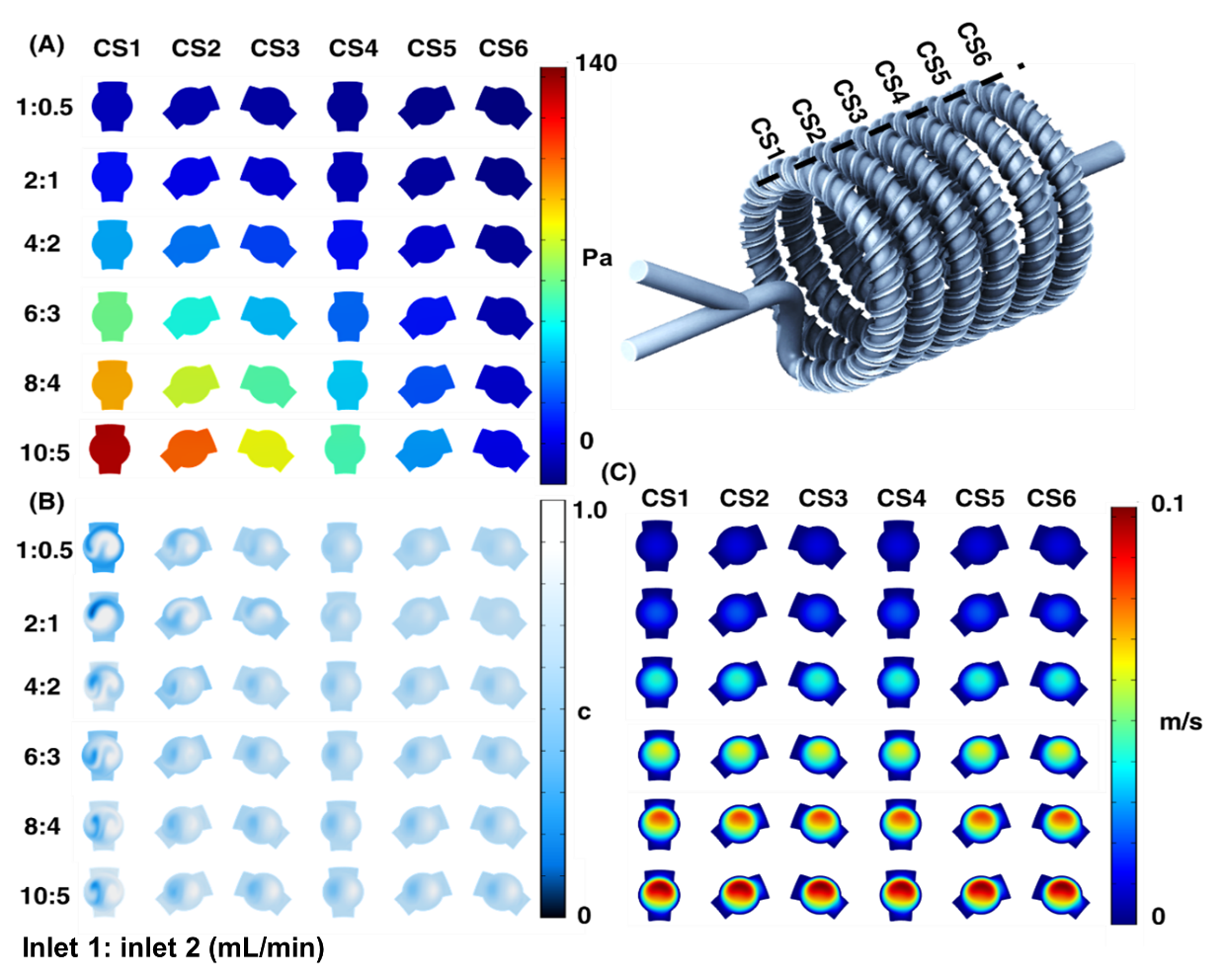


**Fig. S3.** The simulation results of the micromixer demonstrating A) a constant pressure drop across different flow rate. B) The different mixing strategies at different flow rate, and the mixing of different species across the channel, and C) the velocity profile across the channel.

**Fig. S4.** The mixing index and pressure drop of the micromixer at different flow rates. The results showed that the mixing index was as high as 95% when the micromixer was operated at a total flow rate of 3 mL/min.


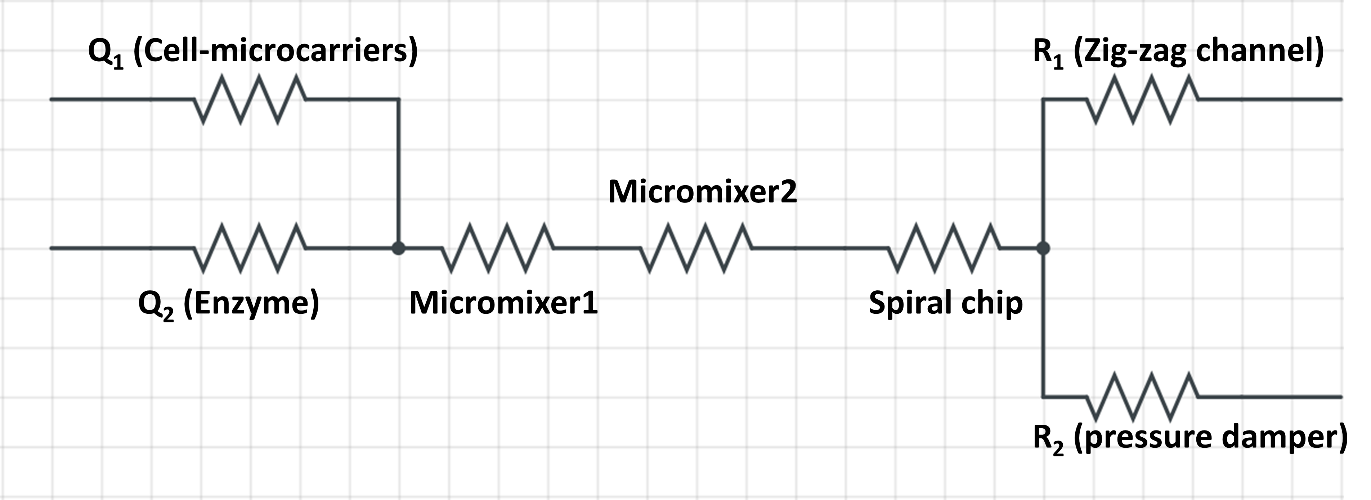


**Fig. S5**. Electric circuit analogy of our system. The serial connection of devices ensures the flow rate was constant in every device. The flow rate is controlled by Q_1_+Q_2_.


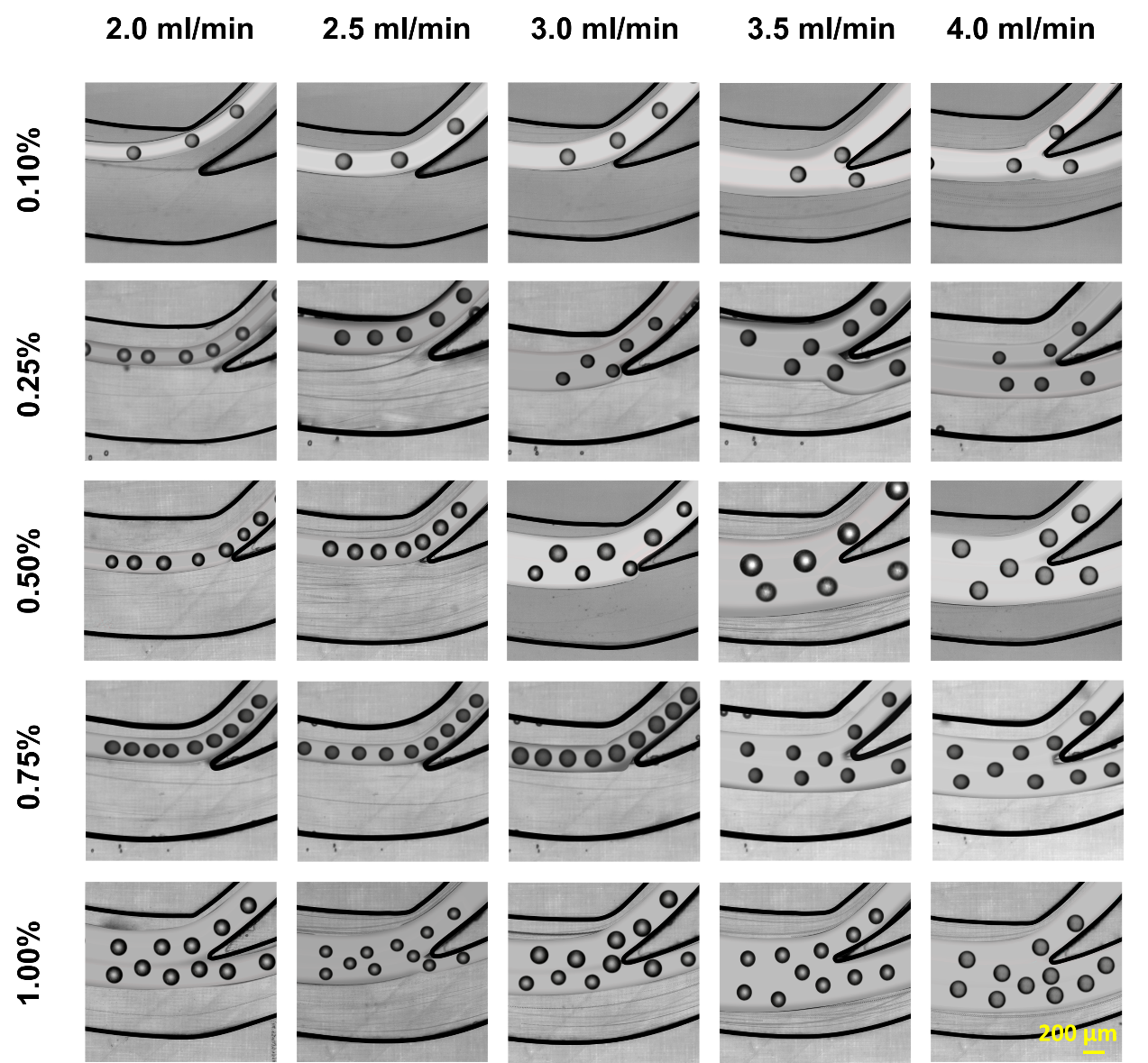


**Fig. S6**. Spiral device characterization with different concentration of microcarriers under different flow rates. The results showed that when the concentration reached 1% the microcarriers cannot be focused on the inner wall. And the flow rate above 3.5 mL/min cause the particles to disperse even under low concentration. The images were made by stacking the video with Z project, standard-deviation mode in ImageJ to visualise the focusing bands, and the microcarriers were added manually later to represent the concentrations and trajectories.


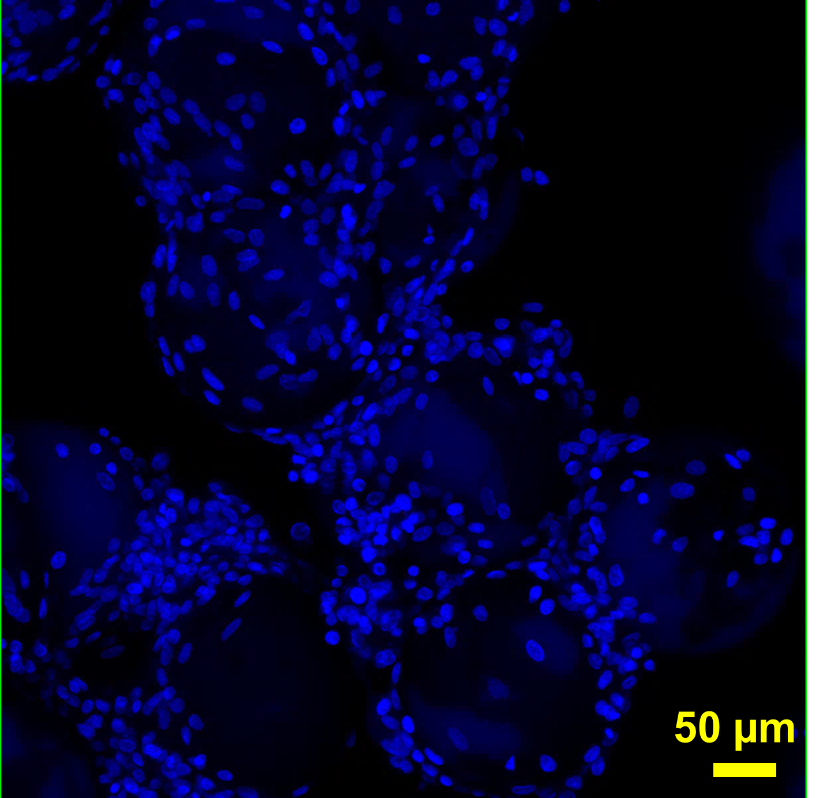


**Fig. S7.** Confocal microscopy images of aggregated microcarriers before cell harvesting.

**
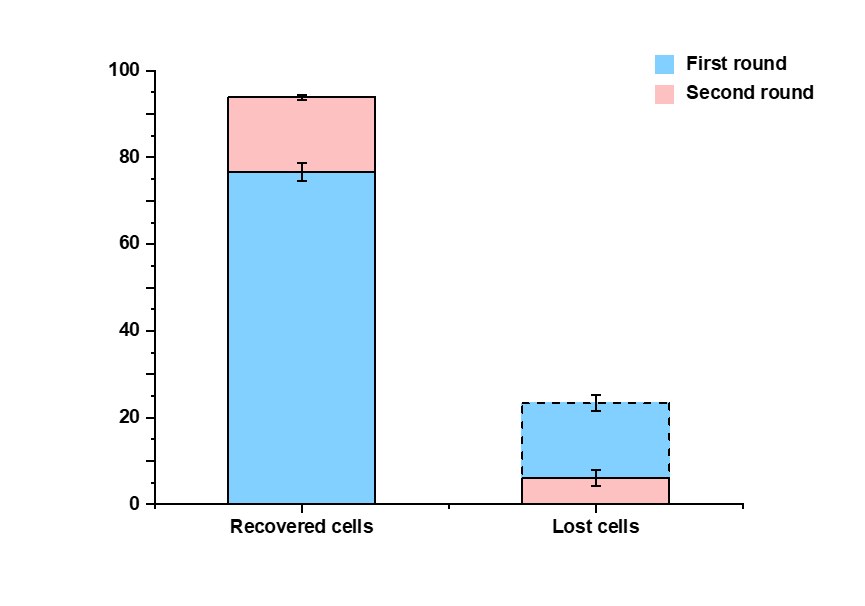
**

**Fig. S8.** The total recovery rate of first and second round purification add-up to be 98.83% in total. The blue dashed area of the lost cells was the cells recovered by the second-round separation.


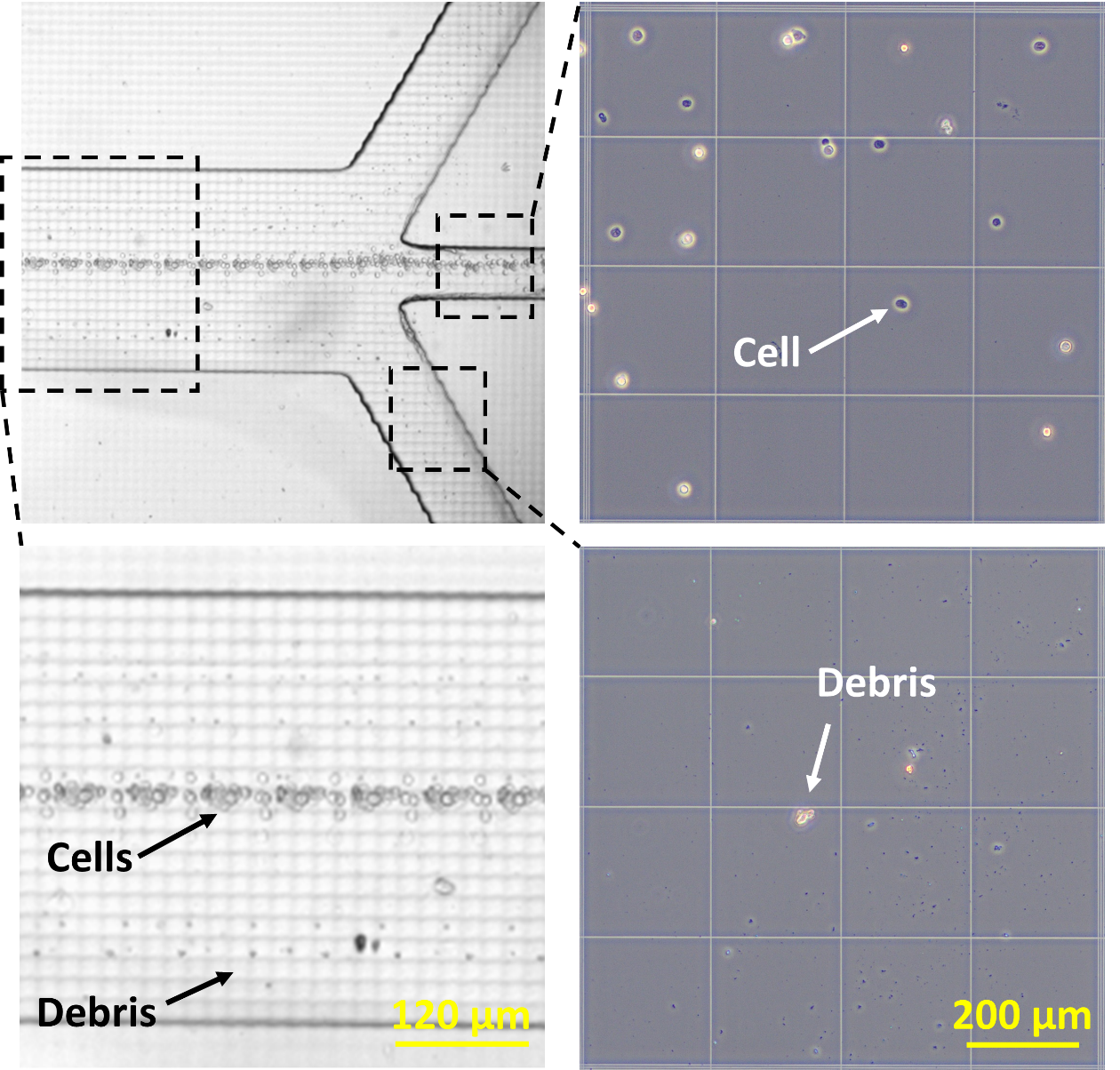


**Fig. S9.** The concentrating results of MSCs in the zigzag channel. The results showed that the cells were concentrated for about 4.5 times, and the recovery rate was about 99.9%.


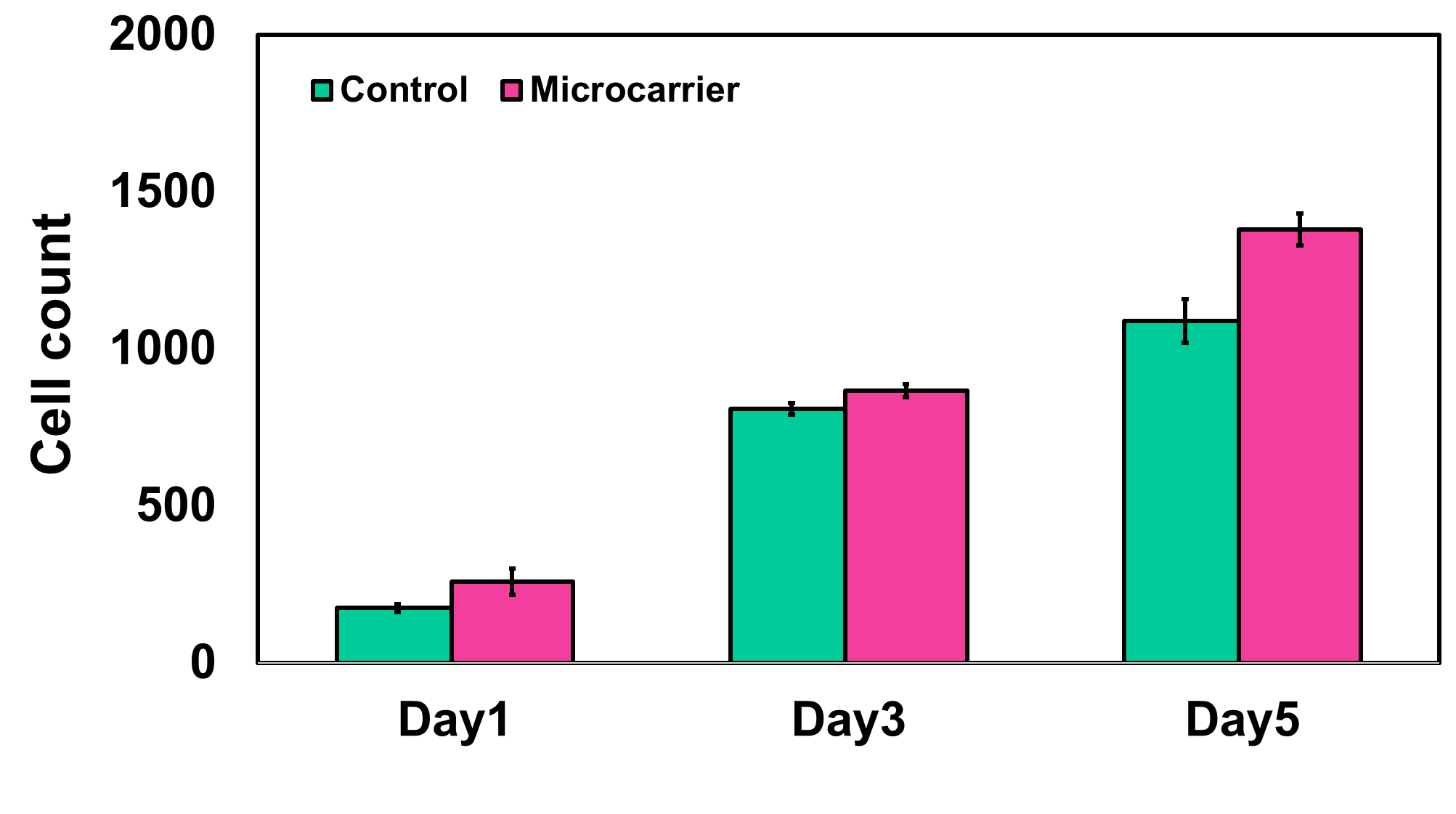


**Fig. S10.** Comparison of the cell attachment after harvesting by counting the number of cells in the pictures in ImageJ.

**References**

1. Moloudi, R., et al., *Scaled‐up Inertial Microfluidics: Retention System for Microcarrier‐based Suspension Cultures.* Biotechnology journal, 2019. **14**(5): p. 1800674.

2. Moloudi, R., et al., *Inertial-Based Filtration Method for Removal of Microcarriers from Mesenchymal Stem Cell Suspensions.* Sci Rep, 2018. **8**(1): p. 12481.

3. Kwon, T., et al., *Microfluidic Cell Retention Device for Perfusion of Mammalian Suspension Culture.* Sci Rep, 2017. **7**(1): p. 6703.

4. Gupta, P., et al., *Optimization of agitation speed in spinner flask for microcarrier structural integrity and expansion of induced pluripotent stem cells.* Cytotechnology, 2016. **68**(1): p. 45-59.

5. Cai, G., et al., *A review on micromixers.* Micromachines, 2017. **8**(9): p. 274.

6. Nguyen, N.-T. and Z. Wu, *Micromixers—a review.* Journal of micromechanics and microengineering, 2004. **15**(2): p. R1.

7. Capretto, L., et al., *Micromixing within microfluidic devices*, in *Microfluidics*. 2011, Springer. p. 27-68.

8. Langille, S.E., *Particulate matter in injectable drug products.* PDA J Pharm Sci Technol, 2013. **67**(3): p. 186-200.

9. Viktorov, V., M.R. Mahmud, and V. Carmen, *Numerical Analysis of Fluid Mixing in Three Split and Recombine Micromixers at Different Inlets Flow Rate Ratio.*

10. Fardjahromi, M.A., et al., *Mussel inspired ZIF8 microcarriers: a new approach for large-scale production of stem cells.* 2020. **10**(34): p. 20118-20128.

11. Cha, J., et al., *A highly efficient 3D micromixer using soft PDMS bonding.* Journal of micromechanics and microengineering, 2006. **16**(9): p. 1778.

12. Xiang, N., et al., *A Multilayer Polymer-Film Inertial Microfluidic Device for High-Throughput Cell Concentration.* Anal Chem, 2019. **91**(8): p. 5461-5468.

13. Taheri, R.A., V. Goodarzi, and A. Allahverdi, *Mixing Performance of a Cost-effective Split-and-Recombine 3D Micromixer Fabricated by Xurographic Method.* Micromachines, 2019. **10**(11): p. 786.

14. Bazaz, S.R., et al., *Obstacle-free planar hybrid micromixer with low pressure drop.* 2020. **24**(8): p. 1-15.

15. Riche, C.T., et al., *Flow invariant droplet formation for stable parallel microreactors.* Nature communications, 2016. **7**(1): p. 1-7.

16. Kang, H., *5. J. Lee, IK Ko, C. Kengia, JJ Yoo, A. Atala.* Nat. Biotechnol, 2016. **34**: p. 312-319.

17. Rafeie, M., et al., *An easily fabricated three-dimensional threaded lemniscate-shaped micromixer for a wide range of flow rates.* Biomicrofluidics, 2017. **11**(1): p. 014108.
